# NrdR in *Streptococcus* and *Listeria* spp.: DNA Helix Phase Dependence of the Bacterial Ribonucleotide Reductase Repressor

**DOI:** 10.1111/mmi.15349

**Published:** 2025-02-18

**Authors:** Saher Shahid, Mateusz Balka, Daniel Lundin, Daniel O. Daley, Britt‐Marie Sjöberg, Inna Rozman Grinberg

**Affiliations:** ^1^ Department of Biochemistry and Biophysics Stockholm University Stockholm Sweden

**Keywords:** allosteric regulation, ATP‐cone, gene expression, ribonucleotide reductase, transcription factor

## Abstract

NrdR is a universal transcriptional repressor of bacterial genes coding for ribonucleotide reductases (RNRs), essential enzymes that provide DNA building blocks in all living cells. Despite its bacterial prevalence, the NrdR mechanism has been scarcely studied. We report the biochemical, biophysical, and bioinformatical characterization of NrdR and its binding sites from two major bacterial pathogens of the phylum *Bacillota* *Listeria monocytogenes* and *Streptococcus pneumoniae* . NrdR consists of a Zn‐ribbon domain followed by an ATP‐cone domain. We show that it forms tetramers that bind to DNA when loaded with ATP and dATP, but if loaded with only ATP, NrdR forms various oligomeric complexes unable to bind DNA. The DNA‐binding site in *L. monocytogenes* is a pair of NrdR boxes separated by 15–16 bp, whereas in *S. pneumoniae* , the NrdR boxes are separated by unusually long spacers of 25–26 bp. This observation triggered a comprehensive binding study of four NrdRs from *L. monocytogenes* , *S. pneumoniae* , *Escherichia coli* , and *Streptomyces coelicolor* to a series of dsDNA fragments where the NrdR boxes were separated by 12–27 bp. The in vitro results were confirmed in vivo in *E. coli* and revealed that NrdR binds most efficiently when there is an integer number of DNA turns between the center of the two NrdR boxes. The study facilitates the prediction of NrdR binding sites in bacterial genomes and suggests that the NrdR mechanism is conserved throughout the bacterial domain. It sheds light on RNR regulation in *Listeria* and *Streptococcus*, and since NrdR does not occur in eukaryotes, opens a way to the development of novel antibiotics.

## Graphical abstract

NrdR in *Streptococcus* and *Listeria* spp. regulates the expression of ribonucleotide reductase genes, which produce DNA building blocks. We show that NrdR binds DNA in an ATP/dATP‐dependent manner, and that optimal binding requires an integer number of DNA helix turns between NrdR boxes, with atypically long spacers in *Streptococcus pneumoniae* . These findings reveal a conserved mechanism of NrdR DNA binding and regulation, providing insights for predicting NrdR binding sites and developing novel antibiotics.

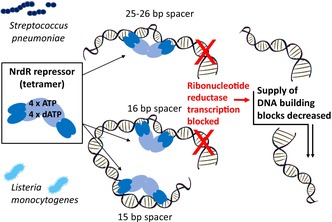

## 1 Introduction

The NrdR protein is a transcriptional repressor consisting of a Zn‐ribbon domain followed by an ATP‐cone (Grinberg et al. 2006, 2009; Torrents et al. 2007). Its acronym (*n*ucleotide *r*e*d*uctase *r*egulator) stems from the fact that it appears to be specific for the essential enzyme ribonucleotide reductase (RNR), which provides living cells with deoxyribonucleotides for DNA synthesis and repair (Mathews 2016, 2018). A balanced supply of deoxyribonucleotides is a prerequisite for high‐fidelity DNA synthesis, and the activity of RNRs is controlled at several levels. All RNRs have a substrate specificity regulation that controls the balance between individual dNTP levels (Hofer et al. 2012). A majority of RNRs also have an activity regulation mediated by an ATP‐cone (Aravind et al. 2000), where binding of ATP activates the enzyme and binding of dATP inhibits the enzyme activity. In class I RNRs, enzyme inhibition is induced by oligomerization involving the ATP‐cone (Martínez‐Carranza et al. 2020; Jonna et al. 2015). Recently, inhibition of the class III RNR enzyme activity was shown to be mediated by increasing the flexibility of a C‐terminal domain and preventing substrate binding (Bimai et al. 2024).

Most bacterial RNRs and a few archaeal RNRs are transcriptionally controlled by NrdR. In *Streptomyces coelicolor (S. coelicolor)* NrdR, the binding of ATP to the ATP‐cone restricts the repressor from binding to specific NrdR binding sites in the promoter regions of RNR operons. With increasing concentrations of dATP, NrdR becomes loaded with both ATP and dATP, which enables the binding of NrdR to DNA (Rozman Grinberg et al. 2022). It is interesting that the same nucleotide binding domain is utilized by both RNRs and their specific repressor NrdR to monitor the physiological balance of nucleotide pools. The NrdR binding site consists of a pair of 16‐bp palindromic sequences (NrdR boxes) (Rodionov and Gelfand 2005) separated by a spacer region, where each half of a palindrome binds one Zn‐ribbon domain. The structure of the functional NrdR tetramer from *S. coelicolor* loaded with four dATP plus four ATP molecules and bound to double‐stranded DNA (dsDNA) containing two NrdR boxes was recently solved to high resolution and revealed a constrained DNA helix with sharp kinks at each NrdR box (Rozman Grinberg et al. 2022).

In this report, we have studied the RNR‐specific transcriptional repressor NrdR from two human pathogens of the phylum *Bacillota*: *Streptococcus pneumoniae (S. pneumoniae)* and *Listeria monocytogenes. S. pneumoniae* is a major global health burden and a leading cause of death among young children, elderly, and immunocompromised persons (Li et al. 2023). *L. monocytogenes* is the third leading cause of death from foodborne illnesses in the United States and a particularly dangerous pathogen among vulnerable groups, such as newborns, pregnant women, and the elderly (Rogalla and Bomar 2023). Evidence exists for *L. monocytogenes* and *S. pneumoniae* RNRs being regulated by NrdR (Nikparvar et al. 2021; Furi et al. 2019); however, the mechanism has not been examined. Only two NrdR representatives have been well characterized before: one from *S. coelicolor* (Grinberg et al. 2006, 2009, Rozman Grinberg et al. 2022) and the other from *Escherichia coli* (Torrents et al. 2007; McKethan and Spiro 2013; Dreux et al. 2015; Naveen and Hsiao 2016). All four bacteria encode two or three different RNR operons, and in three of them ( *L. monocytogenes* , *S. coelicolor* , and *E. coli* ), the spacing between the two NrdR boxes upstream of the operons is 15–16 bp, whereas in *S. pneumoniae* , the spacing between the predicted boxes upstream of both operons is surprisingly 25–26 bp. This observation raises the questions of what constitutes a valid NrdR binding site and how similar the mode of RNR regulation by NrdR is in different bacteria. We explored the mechanism of action of the *Bacillota* NrdR in detail and compared the binding site requirements of NrdRs from four bacteria from three different phyla. Using a set of synthetic dsDNA fragments with a pair of consensus NrdR boxes with spacers between 12 and 27 bp, we show that all NrdR proteins can bind to motifs with 14–17 bp spacers as well as motifs with 24–27 bp spacers, whereas binding to motifs with other spacer lengths is two orders of magnitude less efficient.

## 2 Results

### 2.1 Atypical Organization of NrdR Binding Sites in RNR Promoter Regions of *S. pneumoniae* and *L. monocytogenes*

**FIGURE 1:**
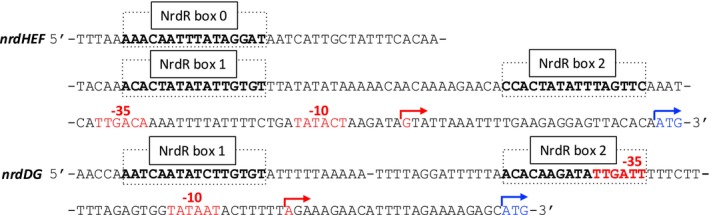
Promoter regions of *nrdHEF* and *nrdDG* in *Streptococcus pneumoniae* . The boldface black/red and boxed regions refer to NrdR boxes 0, 1, and 2 of the *nrdHEF* operon, and NrdR boxes 1 and 2 of the *nrdDG* operon. Red letters and arrows indicate the predicted −35 and −10 regions and the transcription start sites, and blue letters and arrows show translation start sites (Slager et al. 2018). The ATG start codon is 68 bp downstream of Box 2 in the *nrdHEF* promoter region and 53 bp downstream of Box 2 in the *nrdDG* promoter region.

The human pathogend *S. pneumoniae* (Subramanian et al. 2019; Weiser et al. 2018) encodes two RNR operons, *nrdHEF* coding for an aerobic class Ib RNR and *nrdDG* coding for an anaerobic class III RNR. Surprisingly, in the promoter region of *nrdDG* the predicted NrdR boxes are separated by a spacer region of 25 bp (Figure 1), that is, approximately one helix turn longer than the common spacer region of 15–16 bp. Box 2 includes the −35 sequence of the RNA polymerase (RNAP) binding site, while the −10 region is downstream of box 2. In the promoter region of the *nrdHEF* operon, we find three NrdR boxes. Boxes 0 and 1 are separated by 24 bp and boxes 1 and 2 by 26 bp (Figure 1). Both the −35 and −10 sequences are downstream of box 2.

**FIGURE 2:**
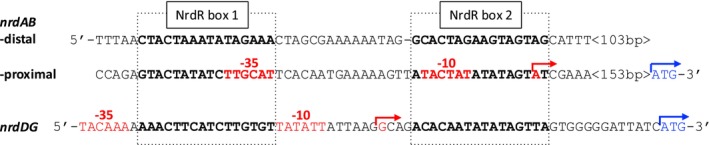
Promoter regions of *nrdABI‐trxL* and *nrdDG* in *L. monocytogenes* . The boldface black/red and boxed regions refer to NrdR boxes 1 and 2. Red letters and red arrows indicate the predicted −35 and −10 regions and the transcription start sites (Mraheil et al. 2011; Wurtzel et al. 2012); blue letters and arrows show translation start sites. The ATG start codon is 157 bp downstream of proximal Box 2 in the *nrdAB* promoter region and 13 bp downstream of Box 2 in the *nrdDG* promoter region.

The human pathogen *L. monocytogenes* (Radoshevich and Cossart 2018; Koopmans et al. 2023) encodes two RNR operons, *nrdABI‐trxL* (here called *nrdAB*), coding for an aerobic class I RNR, and *nrdDG*, coding for an anaerobic class III RNR (Ofer et al. 2011). Upstream of the *nrdDG* operon is a pair of NrdR boxes that overlap the RNAP binding site and the NrdR boxes are separated by a 16‐bp spacer sequence (Figure 2). Interestingly, the upstream region of the *nrdAB* operon instead includes four NrdR boxes, one proximal pair and one distal pair (Figure 2). The promoter‐distal pair of NrdR boxes is separated by a 15‐bp spacer sequence and the promoter‐proximal pair of boxes is separated by a 16‐bp spacer sequence (Ofer 2010). The proximal NrdR box motif overlaps with the RNAP binding site.

### 2.2 Spacer Length Between Pairs of NrdR Boxes in Upstream Regions of RNR Operons in *Streptococcus* spp. and *Listeria* spp.

**FIGURE 3:**
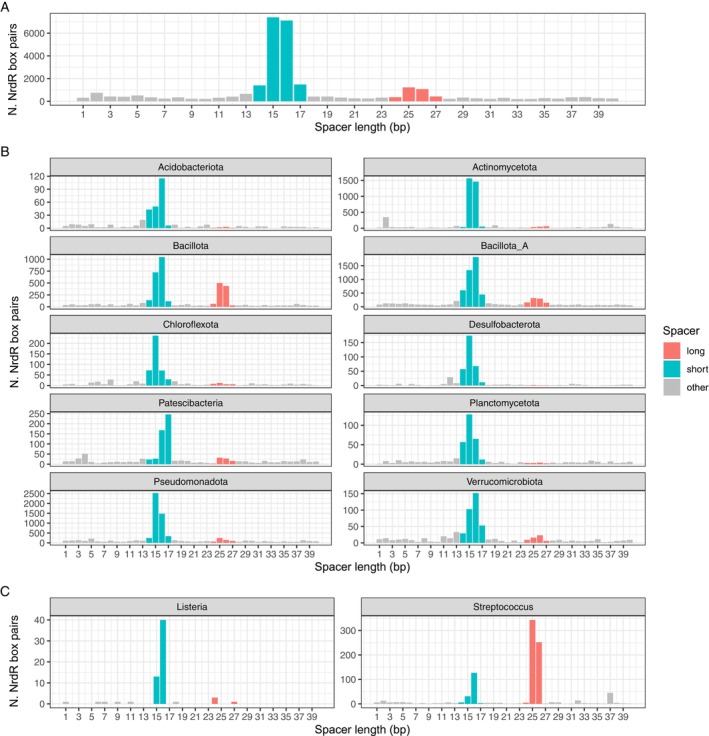
Spacer lengths between two NrdR boxes in RNR promoter regions of (A) all bacteria encoding NrdR, (B) the 10 bacterial phyla with the highest number of NrdR box pairs, and (C) *Listeria* spp., and *Streptococcus* spp. In green: short spacers of 14–17 bp, in red: long spacers of 24–27 bp. Other spacer lengths are in grey. Search for NrdR box pairs was performed as described in Section 4.

The NrdR binding site in most bacteria has a short spacer (15–16 bp) between the two NrdR boxes (Figure 3A). Interestingly, the most common number of base pairs between the two NrdR boxes varied across different bacterial phyla (Figure 3B). A bioinformatic analysis of NrdR box motifs in RNR promoter regions in *Streptococcus* spp. and *Listeria* spp. revealed that most *Streptococci* have long spacers (25–26 bp) between the NrdR boxes, with approximately one‐fifth, including *Streptococcus thermophilus* , having short spacers. The majority of *Listeria* spp. have short spacers (Figure 3C).

**FIGURE 4:**
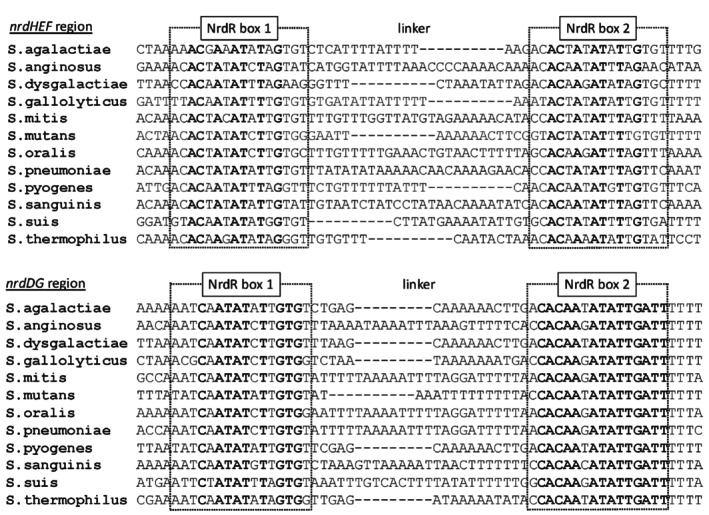
NrdR boxes upstream of *nrdHEF* genes and *nrdDG* genes in selected *Streptococci*. Conserved positions in the box regions are in boldface.

Most *Streptococcus* species had comparable spacer lengths in both promoters, that is, species with a long spacer between boxes in *nrdHEF* promoter also had a long spacer in *nrdDG* promoter and those with a short spacer in *nrdHEF* promoter had a short spacer in *nrdDG* promoter (Figure 4). There were, however, some exceptions, for example, *Streptococcus suis* , with a short spacer in *nrdEF* promoter and a long spacer in *nrdDG* promoter.

### 2.3 Binding of NrdR to the Promoter Regions of RNR Operons in *S. pneumoniae* and *L. monocytogenes*

We used microscale thermophoresis (MST), a technique allowing for the precise determination of the dissociation constants (*K*_D_) of NrdR binding to DNA. Previously, electrophoretic mobility shift assay (EMSA) was used to study NrdR binding to DNA (Grinberg et al. 2006, 2009; Torrents et al. 2007; Case et al. 2011; McKethan and Spiro 2013; Naveen and Hsiao 2016). However, only qualitative results were obtained with EMSA, likely due to the tendency of NrdR to form oligomers, its requirement of nucleotide effectors, and the dissociation of NrdR from DNA during the course of electrophoresis.

*S. pneumoniae* NrdR (SpnNrdR) loaded with dATP and ATP binds comparatively weakly to dsDNA fragments corresponding to the long NrdR box motifs upstream of the *nrdDG* operon and the *nrdHEF* (boxes 1 and 2) operon from *S. pneumoniae* (Table 1; Figure S1A). NrdR boxes in *nrdDG* promoter and boxes 1 and 2 in *nrdEF* promoter are conserved in *Streptococcus* spp. (Figure 4), while box 0 in *nrdEF* promoter is not conserved (data not shown). dATP‐plus ATP‐loaded SpnNrdR binds even less well (*K*_D_ > 7 times higher) to the NrdR boxes 0 and 1 upstream of the *nrdHEF* promoter region, suggesting that this pair of boxes is not recognized in vivo.

**TABLE 1:** Effects of nucleotides on binding of SpnNrdR to RNR promoter regions from *Streptococcus pneumoniae*.

Nucleotide(s)	*nrdHEF* box 0 + 1 (24 bp spacer)	*nrdHEF* box 1 + 2 (26 bp spacer)	*nrdDG* (25 bp spacer)
	*K*_D_ ^a^ (μM)	*K*_D_ ^a^ (μM)	*K*_D_ ^a^ (μM)
dATP + ATP	> 69	11 ± 0.9	15 ± 3
ATP	No binding	> 91	> 3000

^a^ Determined using MST as described in Section 4. The titration curves are presented in Figure S1A. *K*_D_ ± standard deviation was calculated using fits from at least three individual titrations. In cases where *K*_D_ ≥ 69 μM, *K*_D_ is an estimate, because the titration curve did not reach a plateau.

Interestingly, dATP‐plus ATP‐loaded SpnNrdR binds approximately 13 times stronger to the shorter NrdR box motifs in *S. thermophilus* , with a spacer distance of 16 bp, upstream of the *nrdHEF* and *nrdDG* operons (Table 2; Figure S1B). As expected, ATP‐loaded SpnNrdR has extremely low affinity to all dsDNA fragments tested (Tables 1 and 2; Figure S1).

**TABLE 2:** Binding of SpnNrdR in the presence of dATP + ATP to RNR promoter regions from *Streptococcus thermophilus*.

NrdR binding site	SpnNrdR + dATP + ATP	SpnNrdR + ATP
	*K*_D_ ^a^ (μM)	*K*_D_ ^a^ (μM)
*S. thermophilus nrdHEF* NrdR boxes (16 bp spacer)	0.80 ± 0.25	> 42
*S. thermophilus nrdDG* NrdR boxes (16 bp spacer)	1.15 ± 0.32	> 80

^a^ Determined using MST as described in Section 4. Titration curves are presented in Figure S1B. *K*_D_ ± standard deviation was calculated using fits from at least three individual titrations. In cases where *K*_D_ ≥ 42 μM *K*_D_ is an estimate, because the titration curve did not reach a plateau.

*L. monocytogenes* NrdR (LmoNrdR) loaded with dATP and ATP can bind to dsDNA fragments from all three sets of NrdR boxes (*nrdAB* proximal and distal and *nrdDG*) with extremely high affinity (low nM range). Addition of other nucleotides resulted in significantly lower binding affinities or no binding at all (Table 3; Figure S2A) and LmoNrdR did not bind DNA that lacked NrdR boxes (Figure S2). The secondary metabolite di‐cyclic‐AMP (c‐di‐AMP) was earlier reported to bind LmoNrdR (Sureka et al. 2014), but neither c‐di‐AMP nor its combination with ATP or dATP promoted the high‐affinity binding of NrdR to DNA in our experiments (Table 3; Figure S2B). Similarly, *S. coelicolor* NrdR (ScoNrdR) can bind c‐di‐AMP with a *K*_D_ ~3 μM but has low affinity to DNA when loaded with c‐di‐AMP (Rozman Grinberg et al. 2022).

**TABLE 3:** Effects of nucleotides on binding of LmoNrdR to RNR promoter regions from *L. monocytogenes*.

Nucleotide(s)	*nrdAB* proximal (16 bp spacer)	*nrdAB* distal (15 bp spacer)	*nrdDG* (16 bp spacer)
	*K*_D_ ^a^ (nM)	*K*_D_ ^a^ (nM)	*K*_D_ ^a^ (nM)
dATP + ATP	4.7 ± 2	21.2 ± 6	2.2 ± 0.7
ATP	65 ± 23	508 ± 274	209 ± 67
ADP	66 ± 27	271 ± 96	132 ± 45
No effector	> 340	> 1700	> 804
dATP + ADP	10 ± 7	129 ± 47	28 ± 15
dATP	> 909	> 334	79
c‐di‐AMP	169 ± 47	211 ± 98	188
c‐di‐AMP + ATP	39	743	130
c‐di‐AMP + dATP	34	356	201

^a^ Determined using MST as described in Section 4. Titration curves are presented in Figure S2A,B. *K*_D_ ± standard deviation was calculated using fits from at least three individual titrations. In individual cases, one titration was performed for each of the three DNA fragments and approximate *K*_D_s are shown. In cases where *K*_D_ ≥ 334 nM, it is an estimate, because the titration curve did not reach a plateau.

### 2.4 Oligomeric States of *S. pneumoniae* and *L. monocytogenes* NrdR

**FIGURE 5:**
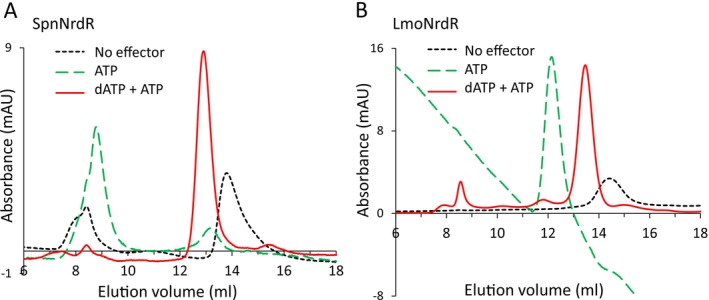
Size exclusion chromatography in the presence of different nucleotide combinations. Representative chromatograms of (A) SpnNrdR and (B) LmoNrdR performed in the presence of ATP (dashed green), dATP + ATP (solid red), and no nucleotides added (dotted black). SEC was performed as described in Section 4. Oligomeric states (Table 4) were estimated using protein elution volume based on a calibration curve derived from globular protein standards.

We performed size exclusion chromatography (SEC) to determine the oligomeric state of SpnNrdR and LmoNrdR in the presence of different nucleotides as described in Section 4. SpnNrdR is an equilibrium of dimers and tetramers in its apo form (in the absence of bound nucleotides), a tetramer in the presence of a combination of dATP and ATP, and predominantly a high‐molecular‐weight oligomer in the presence of ATP (Figure 5A; Table 4). LmoNrdR eluted as a dimer in its apo form, and as a tetramer in the presence of dATP and ATP, and as an octamer in the presence of ATP (Figure 5B; Table 4). LmoNrdR also eluted as a tetramer in the presence of only ADP, a combination of dATP + ADP, or c‐di‐AMP (Figure S3). Notably, ATP‐loaded NrdR, unable to bind DNA, forms different oligomers *in L. monocytogenes* (octamer), *S. coelicolor* (dodecamer), *E. coli* (filaments) and *S. pneumoniae* (large multimers).

**TABLE 4:** Summary of oligomeric states of SpnNrdR and LmoNrdR in the presence of different effectors.

Nucleotides	SpnNrdR				LmoNrdR^a^		
	Peak	MW (kDa)^b^	Number of monomers^c^	Oligomeric state	MW (kDa)^b^	Number of monomers^d^	Oligomeric state
No effector	Major	63 ± 7	3.2	Dimers, tetramers	53 ± 5	2.8	Dimers, tetramers
	Minor	> 800	> 40	Large oligomers			
ATP	Major	≈800	> 40	Large oligomers	152 ± 5	8	Octamers
	Minor	91 ± 8	4.7	Tetramers			
dATP + ATP	Major	108 ± 1	5.5^e^	Tetramers	80 ± 13	4.2	Tetramers

^a^ LmoNrdR oligomeric states were not affected by protein concentration (25–220 μM), pH, or buffer composition (Bis‐Tris propane pH 6.5 and Tris‐Cl pH 8.5).

^b^ Molecular sizes and standard deviations were calculated from at least three individual injections based on a calibration curve as described in Section 4.

^c^ Based on SpnNrdR molecular mass including hexa‐histidine tag of 19.4 kDa.

^d^ Based on LmoNrdR molecular mass including hexa‐histidine tag of 19 kDa.

^e^ Molecular weight of dATP + ATP‐ loaded NrdR complex tends to appear larger on SEC, likely due to its elongated shape (Rozman Grinberg et al. 2022, 2025). We therefore interpret the oligomeric state of dATP+ATP‐SpnNrdR as a tetramer. Since NrdR smallest unit is a dimer interacting via zinc ribbons, the formation of a pentamer is unlikely.

### 2.5 Effect of Spacer Region Length Between the NrdR Boxes on Binding Affinity of NrdR From Four Bacterial Species

**FIGURE 6:**
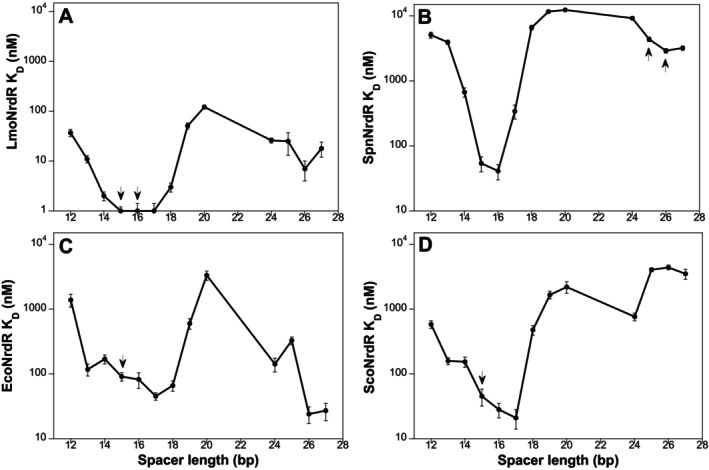
Effect of spacer length in synthetic dsDNA NrdR box pairs on the binding affinity of LmoNrdR (A), SpnNrdR (B), EcoNrdR (C), and ScoNrdR (D) (see Table 5 for details). Arrows denote the spacer lengths of the binding sites in the native RNR upstream regions in each case. The vertical axes are in log scale to facilitate comparisons.

In the *L. monocytogenes nrdAB* promoter region, two sets of RNR boxes are found. In the distal boxes, the spacer between the two boxes is 15 nucleotides, while in the proximal boxes and in the *nrdDG* promoter region, the distance is 16 nucleotides. Our binding results show that LmoNrdR has 5–10 times stronger binding to the *nrdDG* binding site and the *nrdAB* proximal bindings site than it has to the *nrdAB* distal site (Table 3). In the *S. pneumoniae nrdDG* and *nrdHEF* promoters the spacer lengths between NrdR boxes are 25–26 bp, whereas the related *S. thermophilus nrdDG* and *nrdHEF* promoters have a spacer length of 16 bp, and paradoxically bind SpnNrdR 13 times stronger than do the homologous binding sites in *S. pneumoniae* (Tables 1 and 2). We therefore decided to explore the effect of the spacer length on NrdR binding affinity using synthetic dsDNA fragments containing two identical NrdR boxes, designed based on the consensus sequence (Rozman Grinberg et al. 2022), separated by a synthetic spacer region (12–27 bp in length; Tables S1 and S2). The results demonstrate that LmoNrdR is bound tightly to NrdR‐boxes with a spacer region between 14 and 18 bp with *K*_D_s between 1 and 3 nM but binds significantly weaker to shorter or longer spacers (*K*_D_s between 11 and 122 nM) (Figure 6A; Table 5; Figure S4). Interestingly, SpnNrdR appears to have a stricter spacer length requirement with *K*_D_s between 41 and 55 nM for spacers of 15–16 bp (Figure 6B; Table 5; Figure S5).

**TABLE 5:** Binding affinities of NrdRs from *L. monocytogenes* , *Streptococcus pneumonia*, *E. coli* , and *Streptomyces coelicolor* to synthetic NrdR boxes having different spacer lengths between the boxes.

Spacer region (bp)	*K*_D_ ^a^ (nM)			
	LmoNrdR	SpnNrdR	EcoNrdR	ScoNrdR
12	37 ± 6	5057 ± 511	1381 ± 309	580 ± 79
13	11 ± 1.8	3936 ± 290	117 ± 24	158 ± 19
14	2 ± 0.5	699 ± 109	169 ± 27	154 ± 28
15	1 ± 0.2	55 ± 14	91 ± 14	45 ± 13
16	1 ± 0.4	41 ± 11	82 ± 22	28 ± 7
17	1 ± 0.4	340 ± 83	45 ± 6	21 ± 7
18	3 ± 0.6	6603 ± 536	66 ± 12	476 ± 80
19	51 ± 7	11,608 ± 340	601 ± 113	1658 ± 221
20	122 ± 10	12,330 ± 388	3322 ± 540	2191 ± 449
24	26 ± 3	9200 ± 374	141 ± 34	763 ± 104
25	25 ± 12	4371 ± 343	326 ± 44	4051 ± 272
26	7 ± 3	2906 ± 225	24 ± 7	4404 ± 355
27	18 ± 6	3194 ± 238	27 ± 8	3496 ± 626

^a^ Determined using MST as described in Section 4. Titration curves are presented in Figures S4, S5, S7, and S8. *K*_D_ ± standard deviation was calculated using fits from at least three individual titrations.

We also tested binding of the NrdR proteins from *E. coli* (EcoNrdR) and *S. coelicolor* (ScoNrdR) to the set of synthetic NrdR boxes with varying spacer lengths. The four NrdR proteins have 39%–52% sequence identity (Figure S6). For EcoNrdR, good binding was observed for spacers of 15–18 bp with *K*_D_s between 45 and 91 nM (Figure 6C; Table 5; Figure S7), and for ScoNrdR best binding was obtained for spacers of 15–17 bp with *K*_D_s between 21 and 45 nM (Figure 6D; Table 5; Figure S8).

A spacer length of 15–16 bp would result in approximately one and a half turns of the DNA helix between the NrdR boxes (i.e., approximately three turns of the DNA helix between the center of the two NrdR boxes in the binding site). To study whether the different NrdR proteins would also accept a promoter region with an extra DNA helix turn between the center of the NrdR boxes, we tested binding to synthetic fragments with spacer region lengths between 24 and 27 bp. It is obvious from Table 5 and Figure 6 that EcoNrdR can bind to the DNA fragments with 26 and 27 bp spacers with twofold higher affinity than to fragments with a 17 bp spacer. LmoNrdR and ScoNrdR were less tolerant and had *K*_D_s for the 26 and 24 bp long spacers that were 7 and 36 times worse than the best *K*_D_s that were observed to fragments with optimal short spacers. SpnNrdR turned out to be the least tolerant of the tested NrdRs with a 70‐fold higher *K*_D_s for the 26 bp long DNA fragment compared to its *K*_D_ for the DNA fragment with a 16 bp spacer.

### 2.6 In Vivo Confirmation of Spacer Length Results

**FIGURE 7:**
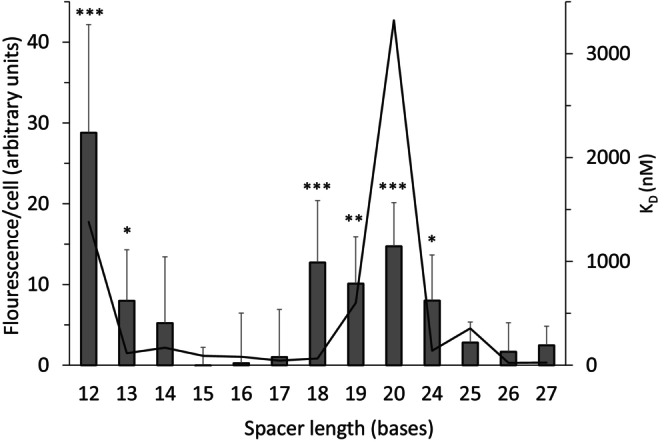
In vivo transcription of synthetic versions of the *E. coli* *nrdHIEF* promoter with varying spacers between NrdR boxes, monitored by using GFP reporter gene. Means and standard deviations, calculated based on at least eight biological replicates are shown. The statistical significance was calculated by the Kruskal–Wallis *H* test and post hoc Dunn's test for multiple comparisons versus a control group, specifically *nrdHIEF* promoter with the wild‐type distance of 15 bp. *p < 0.05, **p < 0.005, ***p < 0.001. For comparison, the *K*_D_ curve of in vitro binding of EcoNrdR to synthetic dsDNA fragments from Figure 6C is included. A reporter plasmid with two mutated NrdR boxes at the wild‐type distance had a fluorescence/cell of 117 ± 66. A reporter plasmid with two wild‐type NrdR boxes at the wild‐type distance in a *nrdR* deletion mutant had a fluorescence/cell of 51 ± 4 calculated based on three biological replicates (see Section 4 for details).

To assess the relevance of the in vitro results of different spacer lengths, we designed a set of experiments in *E. coli* cells. Reporter plasmids with the coding sequence for the green fluorescent protein (GFP) were linked to the upstream region of the *E. coli nrdHIEF* RNR operon. During exponential growth, the *nrdHIEF* operon is strongly repressed by the transcription factors NrdR and Fur, and it is derepressed at stationary growth and/or iron starvation (Torrents et al. 2007; Martin and Imlay 2011). The expression of GFP will therefore be low in our reporter system if NrdR can bind to the NrdR boxes. Expression will be higher if NrdR is prevented from binding by an unfavorable spacing between the NrdR boxes or if the NrdR boxes are mutated. Figure 7 shows that the reporter gene is efficiently repressed when the distance between the NrdR boxes is 15–17 bp, and also repressed at a distance of 26 ± 1 bp. In contrast, the reporter gene is derepressed when the distance between the NrdR boxes is 13 bp and shorter and between 18 and 24 bp. Some degree of repression was evident at all spacer lengths with gene expression reaching a maximum of 22% for a 12 bp spacer compared to a mutant, in which the NrdR boxes were mutated and the in vitro *K*_D_ is ≥ 350 times higher compared to the wild‐type binding site (Rozman Grinberg et al. 2022). In general, our in vivo results (Figure 7) are in good agreement with the in vitro results obtained with the synthetic dsDNA fragments (Figure 6C; Table 5).

### 2.7 Two NrdR Boxes Are Required for High‐Affinity Binding

To determine whether NrdR can bind to individual NrdR boxes, we mutated one box at a time in 57 bp oligonucleotides containing the *E. coli* *nrdHIEF* promoter sequence. While EcoNrdR bound with high affinity to an oligonucleotide containing two wild‐type NrdR boxes, its ability to bind oligonucleotides with only one intact NrdR box was severely impaired, showing 530‐ to 1000‐fold lower affinities respectively for oligonucleotides in which NrdR box 1 and NrdR box 2 were mutated (Table 6; Figure S9).

**TABLE 6:** Binding of EcoNrdR to wild‐type and mutated NrdR boxes derived from the *E. coli* *nrdHIEF* promoter.

NrdR boxes	K_D_ ^a^ (nM)
Wild type	130 ± 25
NrdR box 1 duplicated	213 ± 40
NrdR box 2 duplicated	99 ± 33
NrdR box 1 mutated	≥ 69,000
NrdR box 2 mutated	≥ 130,000

^a^ Determined using MST as described in Section 4. Titration curves are presented in Figure S9. *K*_D_ ± standard deviation was calculated using fits from three individual titrations. In cases where *K*_D_ ≥ 69,000 nM it is an estimate, because the titration curve did not reach a plateau.

Oligonucleotides containing a duplication of either NrdR box 1 or NrdR box 2 of the *nrdHIEF* promoter exhibited high‐affinity binding, with comparable but not identical *K*_D_ values (Table 6; Figure S9). These results indicate that a pair of NrdR boxes is crucial for high‐affinity binding.

## 3 Discussion

RNR‐specific transcriptional repressor NrdR is present in the majority of bacterial genomes; however, it has only been extensively studied in *S. coelicolor* and *E. coli* (Rozman Grinberg et al. 2022, 2025), raising the question how similar its mode of action is in other microorganisms. In this report, we have studied NrdR from two bacterial pathogens: *L. monocytogenes* and *S. pneumoniae* . The NrdR proteins required the binding of cofactors dATP and ATP to their ATP‐cones to form DNA‐binding tetramers. ATP‐loaded LmoNrdR and SpnNrdR were both unable to bind DNA, but formed oligomers of different sizes, similarly to ATP‐cone containing RNRs, in which the active state is conserved and the inactive is not (Hasan et al. 2021). Taken together with previous reports (Rozman Grinberg et al. 2022, 2025) our results suggest that the NrdR mechanism is conserved throughout the bacterial domain.

A majority of NrdR binding sites upstream of the RNR operons consist of two NrdR boxes separated by 15–16 bp (Figure 3). In the genome of *S. pneumoniae* , as well as in the majority of *Streptococcus* spp., the nrdHEF and nrdDG operons contain NrdR boxes separated by exceptionally long spacers. Surprisingly, SpnNrdR binds 13 times stronger to NrdR boxes from *S. thermophilus* with a 16‐bp spacer length compared to its native spacer length of 25–26 bp. Plausibly, the long spacer between NrdR boxes in *S. pneumoniae* is needed to relieve RNR repression, or other factors present in vivo may promote binding of SpnNrdR to its native binding sites.

In *L. monocytogenes* , the *nrdAB* operon is atypical, because it contains two pairs of NrdR boxes. NrdR binds 5–10 times weaker to the *nrdAB* distal NrdR box pair separated by 15 bp compared to both the *nrdAB* proximal and the *nrdDG* box pairs separated by 16 bp. The role of the distal NrdR box pair is unclear, but probably, a more stringent repression is achieved when NrdR binds to both sites simultaneously and the second site may serve to fine tune the regulation.

Our observations triggered a general study of the spacer length requirements for four different NrdR proteins to synthetic dsDNA fragments where the distance between the two NrdR boxes varied between 12 and 27 bp. The four NrdR proteins stem from the *Actinomycetia* (ScoNrdR), *Bacilli* (LmoNrdR and SpnNrdR), and *Gamma Proteobacteria* (EcoNrdR). In general, the strongest binding for all four NrdR proteins was obtained with a spacer length of 15, 16, or 17 bp. A previously solved structure of ScoNrdR bound to a DNA fragment corresponding to the *nrdRJ* NrdR boxes showed a constrained DNA helix with two sharp 90° kinks each covering one of the boxes to which two monomers of the tetrameric NrdR bound via their Zn‐ribbons (Rozman Grinberg et al. 2022). We were hence expecting a narrow spacer length requirement, but surprisingly all four NrdR proteins tested tolerated a spacer length of 3–4 bp around its preferred box distance. Beyond that, the binding strength decreased rapidly and at its extreme was 300 times weaker compared to the strongest binding. Interestingly, all proteins had another *K*_D_ minimum, that is, better binding, at approximately 24‐ to 27‐bp spacer length (Figure 6), which would correspond to one additional turn of the dsDNA helix compared to the optimal shorter spacer length. These results suggest that the optimal distance between the center of the two boxes in the binding motif is an integer of dsDNA turns; in the common case, three turns, but four turns are also tolerated. The affinity of the *E. coli* NrdR to DNA containing the *nrdHIEF* promoter binding sequence decreased drastically if either box 1 or box 2 were mutated, demonstrating that two NrdR boxes are required for high‐affinity binding to RNR promoters.

The presence of an additional helix turn plausibly releases the constraint on the DNA, resulting in a more relaxed complex, but in order to accommodate the fourth helix turn, the angle between the two NrdR dimers binding to each NrdR box, must adjust. We recently showed that the relative orientation of ATP‐cone domains is significantly different in *S. coelicolor* and *E. coli* NrdRs bound to DNA (Rozman Grinberg et al. 2025). We speculate that non‐conserved sequences in the linker region between the zinc finger and ATP‐cone domains and/or at the C‐termina (Figure S6) determine how flexible an NrdR protein is toward the spacing between NrdR boxes. The linker in *L. monocytogenes* , *E. coli* , and *S. pneumoniae* is seven residues long, but only four residues long in *S. coelicolor* . The former three have therefore more similar binding profiles, despite significant differences in affinity. Notably, flexible interdomain linkers were shown to effectively uncouple DNA‐binding domains from constraints imposed by dimerization in different members of the LacI family of regulators (Spronk et al. 1996; Nagadoi et al. 1995; Kallipolitis and Valentin‐Hansen 2004), and a study of zinc‐finger nucleases established a direct relationship between the accepted spacing between its DNA‐binding sites and a short interdomain linker between the DNA‐binding and nuclease domains (Wilson et al. 2013).

When confirming our in vitro results with in vivo studies using a reporter gene fused to the *E. coli* *nrdHIEF* promoter we observed a general consistency. The partial repression of the operon at all spacer lengths can be attributed to NrdR exhibiting low affinity for DNA fragments with unfavorable spacer lengths but being unable to bind to DNA with mutated NrdR boxes (Rozman Grinberg et al. 2022). Plausibly, this residual affinity to nonoptimal binding sites aids NrdR to search DNA for its cognate binding sites. The presence of the Fur repressor in close proximity to this operon's binding site may also contribute to repression in vivo. This assay can be used in the future to explore the regulation of *E. coli* RNR promoters by NrdR and other transcription factors.

Other transcription factors have been reported to recognize binding sites separated by variable spacer lengths, some in a phase‐of‐the‐helix‐dependent manner (Gaston et al. 1990; Ushida and Aiba 1990; Barber et al. 1993; Jørgensen et al. 1998; Schumacher et al. 2015; Yang et al. 2017). Distance requirements between promoter elements are particularly well studied in sigma factors, where the efficiency of RNAP binding to promoters depends on both the sequence of the binding sites and the spacer between them (Forquet et al. 2022; Yeak et al. 2023). Because DNA is helical, the relative orientations of the −35/−10 binding sites are different in spacers of different lengths and can change in the presence of torsional stress, modulating RNAP activity. Moreover, DNA supercoiling varies throughout the bacterial growth cycle as well as in response to a variety of physiological conditions (Dorman 2019); therefore, the spacer acts as an environmental sensor (Klein et al. 2021; Wang and Syvanen 1992).

Bacteria often cope with a frequently changing environment, and many encode more than one RNR gene (Lundin et al. 2015) with NrdR boxes in their promoter regions that may vary in sequence, spacer length, and position relative to promoter elements (Figures 1 and 2). Hypothetically, NrdR could sense the environmental conditions via the DNA supercoiling state in each RNR promoter and bind differentially to NrdR boxes in response to conditions reflecting a required stringency of RNR regulation. DNA supercoiling and likely also NrdR binding are affected by the presence of RNAP and other regulatory proteins (Dorman 2019; Torrents 2014). NrdR binding and the degree of repression will therefore depend on all the above making RNR regulation a fine‐tuned interplay between NrdR, RNAP, and other transcription factors binding in the region. *P. aeruginosa* NrdR was suggested to regulate the expression of *topA*, encoding DNA topoisomerase I required for the relaxation of negatively supercoiled DNA (Crespo et al. 2015). While this may provide a link between NrdR and DNA topology, the mechanism is questionable, since NrdR presumably acts as an activator rather than a repressor, and there is only one NrdR box in the *topA* promoter. Our results, however, for both *S. coelicolor* (Rozman Grinberg et al. 2022) and *E. coli* NrdR (in vitro and in vivo, this study) suggest that NrdR requires two boxes for binding and is unable to bind a single NrdR box.

Taken together, our results are essential for understanding the general mode of action of NrdR, a universal repressor of bacterial RNR genes. They will facilitate the accurate identification of NrdR binding sites in bacterial genomes and contribute to the development of antimicrobial agents based on NrdR and its RNR regulons in important pathogens.

## 4 Materials and Methods

### 4.1 Plasmids and Reagents

The *nrdR* gene from *L. monocytogenes* serotype 1/2a str, S10403S (LMRG_RS07775, WP_003723246) and the *nrdR* gene from *S. pneumoniae* TIGR4 (SP_1713, AAK75791), both cloned in pET30a(+) plasmid using NdeI and XhoI restriction sites, were ordered from GenScript, resulting in pET30a(+)::Lmo*nrdR and pET30a(+)::SpnnrdR*, containing C‐terminal hexa‐histidine tags. ATP and dATP solutions (100 mM, pH 7.0) were purchased from ThermoFisher Scientific. c‐diAMP was obtained from Jena Bioscience, Germany in lyophilized form and resuspended in water to 10 mM solution. ADP (Sigma Aldrich) powder was dissolved in water to get 100 mM solution and pH was adjusted to 7.0.

### 4.2 Protein Expression and Purification

*E. coli* BL21(DE3) were transformed with the pET30a(+)::Lmo*nrdR* or *pET30a(+)::SpnnrdR*. Single colony of the transformed cells was used to inoculate the LB medium supplemented with kanamycin (50 μg mL^−1^) and grown overnight at 37°C. This overnight culture was used to inoculate LB medium to OD_600_ 0.1 and the cells were allowed to grow under vigorous shaking at 37°C. When OD_600_ of 0.8–0.9 was achieved, the culture media was cooled down on ice and 0.5 mM IPTG and 0.1 mM Zn(CH_3_CO_2_)_2_ were added. The cultures were grown overnight at 20°C and the cells expressing LmoNrdR or SpnNrdR were harvested by centrifugation.

Cell pellet was resuspended in lysis buffer containing 1 M NaCl, 20% glycerol, 10 mM Imidazole, 2 mM dithiothreitol (DTT) and 0.05 M Tris‐Cl pH 8.5 at 4°C for LmoNrdR and 0.05 M HEPES pH 7.2 for SpnNrdR. Phenylmethylsulfonyl fluoride (PMSF) was added to 1 mM concentration to the cell suspension, which was then sonicated in an ultrasonic processor (Misonics) until a clear lysate was obtained. The lysate was centrifuged at 18,000 *g* at 4°C for 45 min and the supernatant was used for protein purification.

For purification, the supernatant was loaded into HisTrap FF Ni‐Sepharose column (Cytiva) which was pre‐equilibrated with lysis buffer (without PMSF). After loading the protein, the column was extensively washed with buffer containing 10 and 60 mM imidazole. The his‐tagged LmoNrdR/SpnNrdR were eluted with elution buffer containing 0.5 M NaCl, 20% glycerol, 0.5 M Imidazole, 2 mM DTT and 0.05 M Tris‐Cl pH 8.5 at 4°C for LmoNrdR and 0.05 M HEPES pH 7.2 for SpnNrdR. The purified protein was then desalted using HiPrep 26/10 Desalting column (Cytiva) equilibrated with storage buffer [0.05 M Tris‐Cl pH 8.5 at 4°C, 0.5 M NaCl, 20% glycerol, 1 mM tris(2‐carboxyethyl)phosphine [TCEP] for LmoNrdR and 0.05 M HEPES pH 7.2 at RT, 0.5 M NaCl, 20% glycerol, 1 mM TCEP for SpnNrdR] and stored at −80°C. Protein concentration was determined using Coomassie Plus protein assay reagent (ThermoFisher Scientific) using a BSA standard curve.

To remove traces of bound nucleotides from LmoNrdR, the purified protein was subjected to hydrophobic interaction chromatography (HIC) as described previously (Rozman Grinberg et al. 2022) using HiTrap Phenyl HP column (Cytiva) in buffer (0.025 M Bis–Tris Propane (pH 6.5), 0.75 M ammonium sulfate and 0.1 mM TCEP). After applying the protein, the column was washed extensively with the same buffer (50 CV) and the protein was eluted in the same buffer without ammonium sulfate. The eluted protein was then desalted using a PD10 column (Cytiva) in buffer containing 0.025 M Bis–Tris Propane pH 6.5, 0.3 M NaCl, 10% glycerol and 1 mM TCEP. Protein concentration and recovery after HIC was 0.8 mg mL^−1^ and ~20%, respectively. The apo‐LmoNrdR was aliquoted and stored in −80°C until used.

### 4.3 Microscale Thermophoresis

MST was used to study the binding of NrdR proteins to strain specific and synthetic NrdR boxes as described previously (Rozman Grinberg et al. 2022). Oligonucleotides of 57–69 bp containing two NrdR boxes, the spacer region between them and flanking regions of 5 bp were ordered from Merck (Germany). The 5′ end of sense strand of each oligonucleotide was labeled with Cyanine 5 dye (Cy5) by the manufacturer, while the antisense strand was unlabeled. Oligonucleotides were either obtained as 100 μM solution in Tris‐EDTA buffer or in lyophilized form and resuspended in 50 mM Tris‐Cl (pH 8.0), 50 mM NaCl and 1 mM EDTA. Single stranded oligonucleotides were annealed by mixing 50 and 57.5 pmoles of labeled and unlabeled oligonucleotides respectively, in 50 μL buffer (10 mM Tris‐Cl pH 8.0 and 50 mM NaCl) using a thermoblock. Annealing program involved incubation for 5 min at 95°C followed by gradual cooling to 25°C using 140 cycles of—0.5°C and 45 s per cycle, resulting in 1 μM double stranded DNA. Integrity of the annealed oligonucleotides was determined by application to Mini‐PROTEAN native 5% polyacrylamide TBE gel (BioRad), staining with SYBR safe DNA gel stain (Thermo Fischer Scientific) and analysis using gel imager system C600 (Azure Biosciences) at wavelengths suitable for detection of Cy5 and Cy3 emission signals (Figure S10). Oligonucleotides used to assay NrdR binding to native *L. monocytogenes* , *S. pneumoniae* and *S. thermophilus* promoters are listed in Table S3. The synthetic (Synt) oligonucleotides with different spacer lengths are listed in Tables S1 and S2 (for sense and antisense oligonucleotides respectively).

MST for analyzing the binding of LmoNrdR and SpnNrdR to strain‐specific NrdR boxes and synthetic oligonucleotides containing NrdR boxes with spacers of different lengths and binding of EcoNrdR to wild‐type and mutated fragments containing NrdR boxes of the *E. coli* *nrdHIEF* promoter was performed using Monolith NT.115 instrument (Nanotemper Technologies, Germany). The binding of ScoNrdR and EcoNrdR to the synthetic oligonucleotides was tested using Monolith NT. Automated instrument (Nanotemper Technologies, Germany).

For LmoNrdR, MST buffer contained 0.025 M Bis–Tris Propane (pH 6.5), 5% glycerol, 100 mM KCl, 5 mM MgCl_2_, 1 mM DTT and 0.05% Tween 20. For assaying binding of LmoNrdR to native NrdR boxes in the presence of different nucleotide effectors, apo‐LmoNrdR, obtained by HIC, was supplemented with the nucleotides of choice. For assaying binding to synthetic oligonucleotides, protein purified by nickel affinity chromatography, supplemented with dATP and ATP was used. Either one nucleotide at 1 mM concentration or two nucleotides at 0.5 mM concentration each were included in MST buffer. To study the binding of LmoNrdR to oligonucleotides containing native NrdR boxes, medium MST power, 90% of excitation power and 2 nM DNA was used. For the analysis of LmoNrdR binding to synthetic oligonucleotides with different spacer lengths, high MST, 95% excitation power and 0.5 nM DNA were used.

For SpnNrdR the buffer was 0.025 M HEPES (pH 7.2), 250 mM NaCl, 15% glycerol, 2.5 mM MgCl_2_, 2 mM DTT, 0.1 mg mL^−1^ BSA, 0.05% Tween 20, 0.5 mM ATP, and/or 0.5 mM dATP. MST was done at medium MST power, 90% excitation power and with 5 nM DNA.

For EcoNrdR, the buffer was 0.025 M Tris‐Cl (pH 8.5 at 4°C), 100 mM NaCl, 7 mM MgCl_2_, 1 mM DTT, 0.025% Tween20, 1 mM ATP, and 1 mM dATP. MST was done at high MST power with 50% excitation power using 10 nM DNA.

For ScoNrdR, the buffer was 0.025 M Tris‐Cl (pH 8.0), 150 mM NaCl, 5% glycerol, 10 mM MgCl_2_, 2 mM DTT, 0.05% Tween 20, 1 mM ATP, and 1 mM dATP. MST was done at medium MST power, 40% excitation power and with 10 nM DNA.

The results were analyzed using the MO.Affinity Analysis v2.3 software (Nanotemper) with default parameters. *K*_D_ and standard deviation were calculated using fits from at least three individual titrations. The measured *K*_D_s reflect the binding of the NrdR tetramer to the NrdR binding site composed of two NrdR boxes. We attempted to analyze separate *K*_D_s for binding to the individual NrdR boxes, but the analysis package could not differentiate between two binding events. In individual cases, the *K*_D_s could not be reliably determined since the titration curves did not reach a plateau or no binding was observed (see titration curves in Supporting Information). The indicated *K*_D_s, derived from these curves, are therefore only estimates.

### 4.4 Analytical Size Exclusion Chromatography

To see the oligomerization states of the protein in the presence or absence of different nucleotides, SEC was done at 4°C using Superdex S200 10/300 Increase or Superdex S200 3.2/300 Increase (Cytiva) attached to an Äkta prime system (Cytiva). Molecular weights were estimated from the calibration curve, which was derived from the globular protein standards using both the high‐ and low‐molecular‐weight SEC marker kits (Cytiva). Standard deviations were calculated using data from at least three runs.

For LmoNrdR protein, the nucleotides/combinations of nucleotides added to the protein included dATP + ATP, ATP, ADP, dATP + ADP, and c‐di‐AMP. The column was equilibrated with 25 mM Bis–Tris Propane (pH 6.5), 5% glycerol, 5 mM MgCl_2_, 100 mM KCl, and 1 mM DTT. One (0.2 mM) or a combination of two nucleotides (0.1 mM each) (ATP, dATP, ADP, and c‐di‐AMP) were included in the buffer. The protein (50 μM) was incubated for 10 min at room temperature with 1 mM nucleotide and 5 mM MgCl, centrifuged and 100 μL sample was injected to the column. Runs of apo‐NrdR were performed without the addition of nucleotides to the protein or buffer.

For SpnNrdR protein, the column was equilibrated with 25 mM HEPES (pH 7.2), 2.5 mM MgCl_2_, 15% glycerol, 250 mM NaCl, and 0.1 mM TCEP and either ATP and dATP 0.1 mM each or 1 mM ATP. SpnNrdR protein (50 μM) was incubated for 10 min at room temperature with either 0.5 mM ATP, 0.5 mM dATP, and 2.5 mM MgCl or 1 mM ATP and 2.5 mM MgCl, centrifuged and 100 μL sample was injected to the column. Runs of apo‐NrdR were performed without addition of nucleotides to protein or buffer.

### 4.5 Promoter Activity Assay for In Vivo Analysis of Repressor Binding

Double‐stranded DNA fragments (201–216 bp) containing the ATG start codon of *E. coli* K‐12 strain MG1655 *nrdHIEF* gene and 143 bases upstream, that is, containing the NrdR and Fur binding sites (P_nrdHIEF_) were ordered from GenTitan Gene Fragments Service (Genscript). The sequence of the region containing the NrdR boxes and the spacer between them in each of the constructs are given in Table S4. The fragments were cloned into the pSEVA631(Sp) [P_ibpA_‐gfp_ASV_] plasmid (Zutz et al. 2021) using the in vivo cloning method (Watson and Garcia‐Nafria 2019). In short, the pSEVA plasmid was amplified by PCR using the Q5 High‐Fidelity DNA Polymerase (New England Biolabs) and the Fwd_GFP_rep_AN (ATGCGTAAAGGAGAAGAACTTTTCAC) and Rev_GFP_rep_AN primers (TTAATTAAAGGCATCAAATAAAACGAAAGG). This PCR removed the region encoding P_ibpA_. The PCR product was digested with DpnI, separated by agarose gel electrophoresis, and purified using DNA gel extraction kit (GeneJET; Thermo Scientific), mixed briefly at room temperature with P_nrdHIEF_ dsDNA fragments (one at a time) and transformed into the chemically competent MC1061 strain (str.K‐12F^−^ λ^−^ Δ(*ara‐leu*)*7697* [*araD139*]B/rΔ(*codB‐lacI*)*3 galK16 galE15* e14^−^ *mcrA0 relA1 rpsL150*(Str^R^) *spoT1 mcrB1 hsdR2*(*r* ^−^ *m* ^+^)). The integrity of the resulting plasmids pSEVA631(Sp) [P_nrdHIEF_‐gfp_ASV_] was verified by sequencing carried out by Eurofins Genomics (Germany). *nrdR* deletion mutant strain from the *E. coli* Keio knockout collection (Baba et al. 2006) was ordered from Horizon Discovery (clone catalog number OEC4987‐213604652), made chemically competent and transformed with pSEVA631(Sp) [P_nrdHIEF_‐gfp_ASV_] plasmid containing NrdR boxes at the wild‐type distance.

A single colony of MC1060 harboring pSEVA631(Sp) [P_nrdHIEF_‐gfp_ASV_] was used to inoculate 500 μL of LB media (Miller) containing 50 μg mL^−1^ spectinomycin. Cultures were grown at 37°C with shaking at 185 rpm for 16 to 20 h. A 50 μL aliquot was back‐diluted into 5 mL of fresh LB containing 50 μg mL^−1^ spectinomycin in a 5 mL 24‐well plate. Cultures were grown at 37°C with shaking at 185 rpm to an OD_600_ of approximately 0.8. Aliquots of 2–5 mL bacterial culture were collected by centrifugation at 3220*g* for 15 min. The culture medium was removed, and the pelleted cells were resuspended in 200 μL of buffer (50 mM Tris–HCl pH 8.0, 200 mM NaCl, 15 mM EDTA). The cell suspension was transferred to a 96‐well optical bottom black‐wall plate (Thermo Scientific) and the fluorescence was determined in a SpectraMax Gemini EM plate reader (Molecular Devices, UK). Fluorescence from pSEVA631(Sp) [P_nrdHIEF_‐gfp_ASV_] was measured with an excitation wavelength of 475 nm and emission wavelength of 515 nm. A long‐pass emission cut‐off filter of 495 nm was used to reduce background. Fluorescence values were normalized by the optical density and the volume of the sample (OD_600_). These values were obtained from a 200 μL aliquot of bacterial culture using a SpectraMax m2e plate reader (Molecular Devices) at 600 nm.

Statistical analysis was performed in SigmaPlot 13. Statistical significance was determined using Kruskal–Wallis test followed by Dunn's post hoc test for multiple comparisons versus a control group, specifically the *nrdHIEF* promoter containing wild‐type spacer of 15 bp between NrdR boxes.

### 4.6 Bioinformatics

After identifying potential RNR operons—genes annotated as RNRs by Prokka (v. 1.14.6, Seemann 2014), separated by no more than 300 nucleotides—in all species representative bacterial genomes in the GTDB (release 08‐RS214, Parks et al. 2018) 500 nucleotides upstream of each operon were extracted. Each fragment was searched with a regular expression for a pair of NrdR boxes separated by a spacer of no more than 50 nucleotides (“C.[AT].[A][AT].[AT].G.{1,50}?C.[AT].[A][AT].[AT].G”). Spacers with a length between 20 and 23 (corresponding to a box distance of 14–17 bp) were considered “short” and those between 30 and 33 (corresponding to a box distance of 24–27 bp) were considered “long”. Finally, each class of lengths was counted.

## Author Contributions

**Saher Shahid:** investigation, visualization, validation. **Mateusz Balka:** investigation, visualization, validation. **Daniel Lundin:** data curation, investigation, formal analysis. **Daniel O. Daley:** funding acquisition, supervision, methodology, validation, resources. **Britt‐Marie Sjöberg:** conceptualization, funding acquisition, writing – original draft, writing – review and editing, visualization, supervision, validation, resources. **Inna Rozman Grinberg:** conceptualization, funding acquisition, writing – original draft, writing – review and editing, visualization, supervision, validation, formal analysis, methodology.

## Conflicts of Interest

The authors declare no conflicts of interest.

## Supporting information


Appendix S1.

## Data Availability

The data underlying this article are available in the article and in its Supporting Information.
