# Supplementary material for: NrdR in Streptococcus and Listeria spp.: DNA Helix Phase Dependence of the Bacterial Ribonucleotide Reductase Repressor
Source: Mol Microbiol. 2025 Feb 18;123(5):406–19. doi: 10.1111/mmi.15349 (PMC12051234; doi:10.1111/mmi.15349)
Supplement: Supplementary file 1 — Appendix S1. [file MMI-123-406-s001.pdf]

Supplementary data

NrdR in *Streptococcus* and *Listeria* spp.: DNA helix phase-dependence of the bacterial ribonucleotide reductase repressor

Saher Shahid, Mateusz Balka, Daniel Lundin, Daniel O. Daley, Britt-Marie Sjöberg and Inna Rozman Grinberg*

Department of Biochemistry and Biophysics, Stockholm University, SE-10691 Stockholm, Sweden

*corresponding author

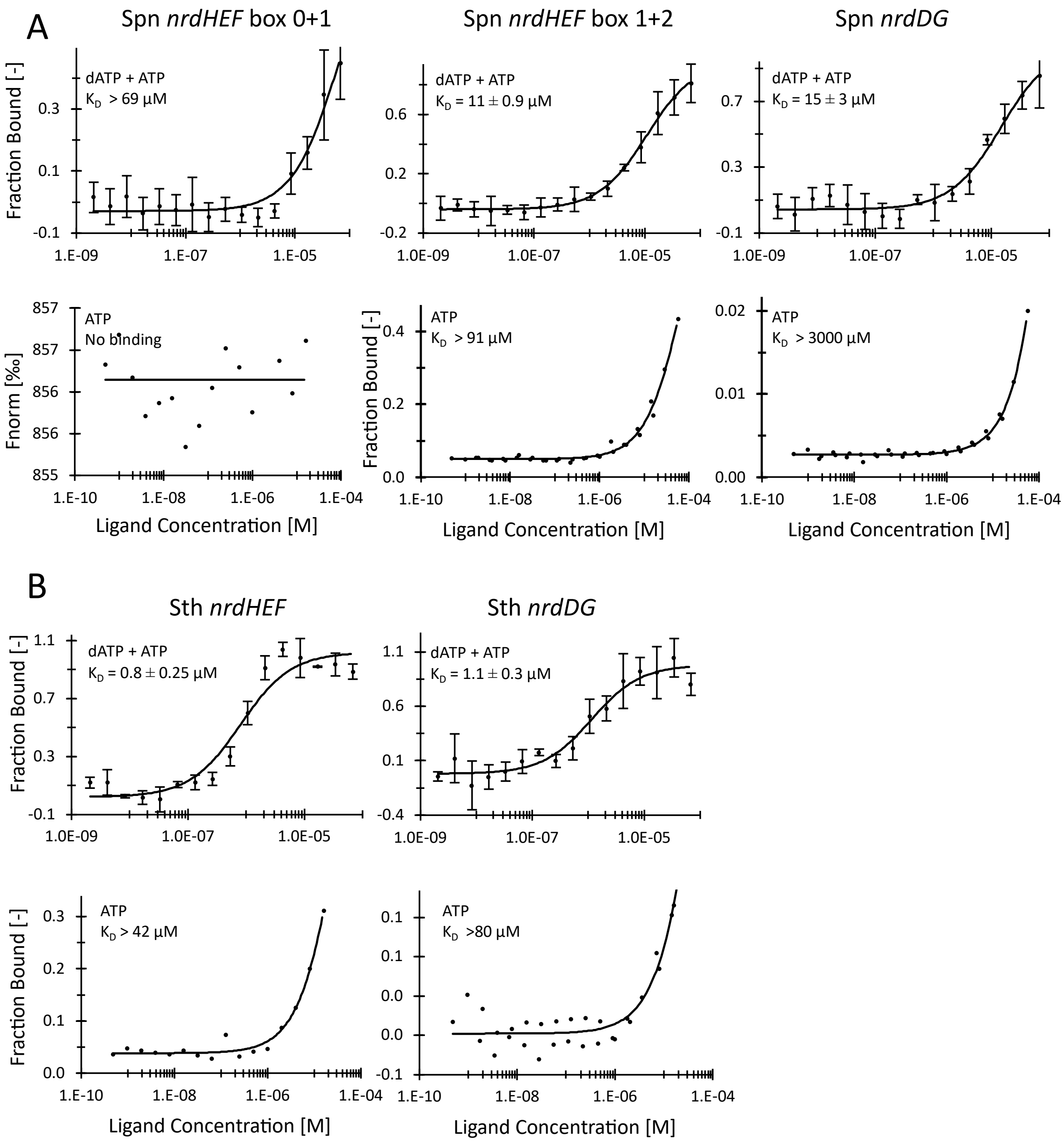

Supplementary figure 1. Binding of *Streptococcus pneumoniae* NrdR to native *nrdHEF* and *nrdDG* promoters (A) and promoters of *Streptococcus thermophilus* (B) in the presence of different nucleotides, determined by MST. Plots of protein fraction bound vs. the concentration of ligand (NrdR) are shown. Lines represent fits of the data points using the K_D fit derived from the law of mass action. K_D and standard deviations (mean values ± SD) were calculated by the analysis software using fits from at least three individual titrations. Flat lines were produced in the cases where no fit could be generated by the software and the parameters therefore were fixed as for non-binder ligands. In these traces the normalized fluorescence Fnorm (‰) from T-Jump and Thermophoresis vs. concentration of ligand are shown. In some cases, the fits resulted in K_Ds in the range of 42 - 3000 µM, but the actual K_D cannot be determined, since the curves do not reach a plateau.

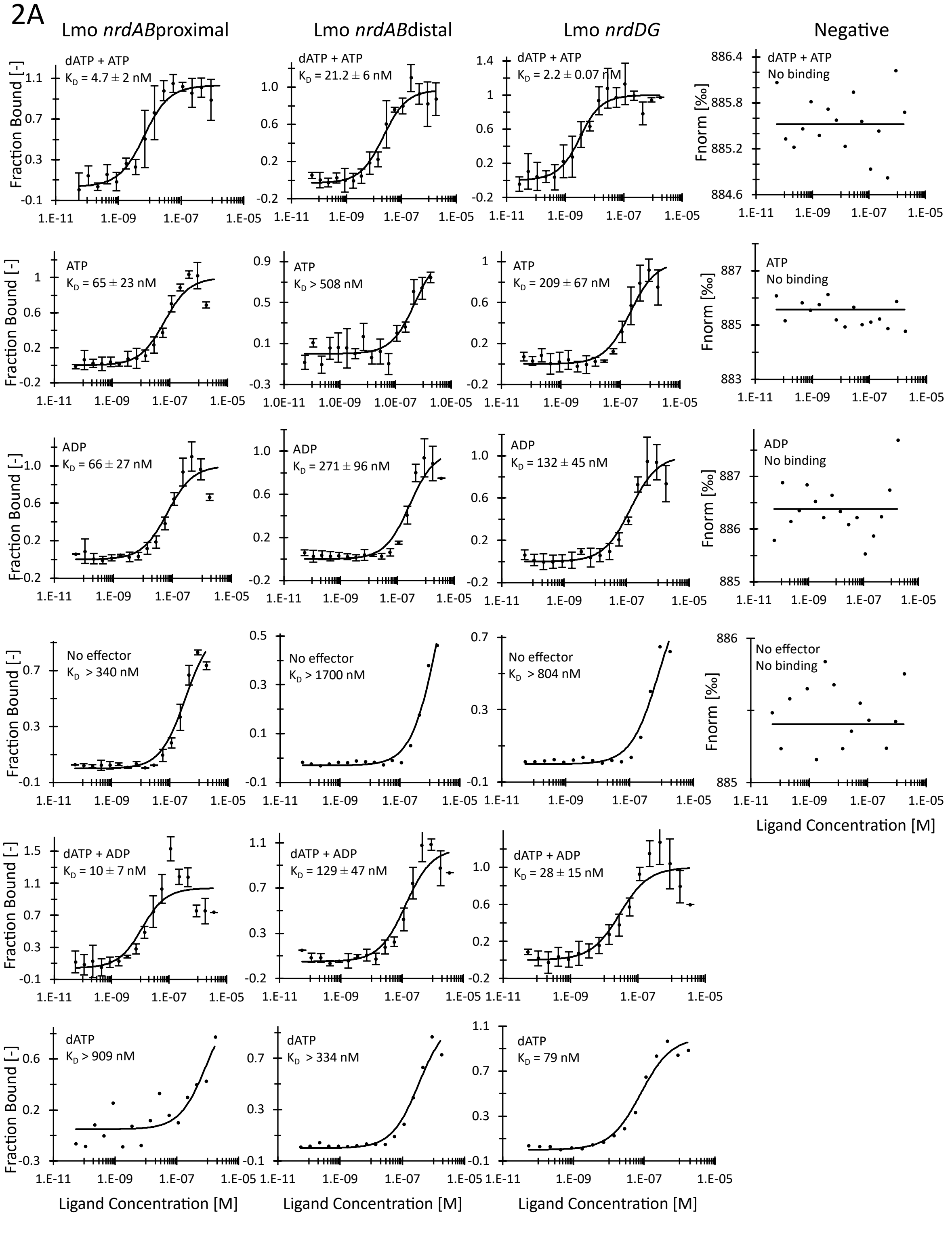

2A
Lmo nrdABproximal
Lmo nrdABdistal
Lmo nrdDG
Negative
dATP + ATP
KD = 4.7 ± 2 nM
KD = 21.2 ± 6 nM
KD = 2.2 ± 0.07 nM
No binding
ATP
KD = 65 ± 23 nM
KD > 508 nM
KD = 209 ± 67 nM
ADP
KD = 66 ± 27 nM
KD = 271 ± 96 nM
KD = 132 ± 45 nM
No effector
KD > 340 nM
KD > 1700 nM
KD > 804 nM
dATP + ADP
KD = 10 ± 7 nM
KD = 129 ± 47 nM
KD = 28 ± 15 nM
dATP
KD > 909 nM
KD > 334 nM
KD = 79 nM
Fraction Bound [-]
Fnorm [‰]
Ligand Concentration [M]

2B

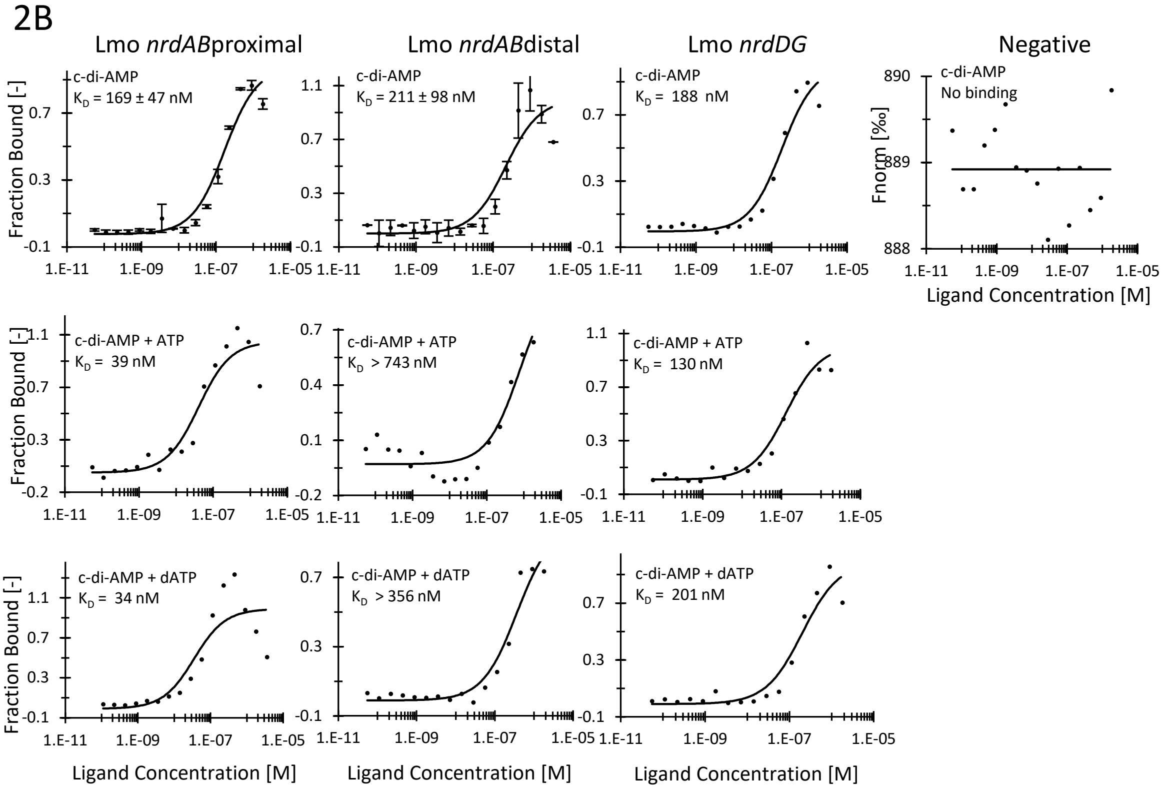

Supplementary figure 2. (A, B) Binding of *L. monocytogenes* NrdR to native promoters in the presence of different nucleotides and their combinations determined by MST. Plots of protein fraction bound vs. the concentration of ligand (NrdR) are shown. Lines represent fits of the data points using the K_D fit derived from the law of mass action. K_D and standard deviations (mean values ± SD) were calculated by the analysis software using fits from at least three individual titrations. Flat lines were produced in the cases where no fit could be generated by the software and the parameters therefore were fixed as for non-binder ligands. In these traces the normalized fluorescence Fnorm (‰) from T-Jump and Thermophoresis vs. concentration of ligand are shown. In some cases, the fits resulted in K_Ds in the range of 334 - 1700 nM, but the actual K_D cannot be determined, since the curves do not reach a plateau. The 'Negative' control DNA sequence is derived from the region upstream of the Lmo *nrdAB* promoter and contains 57 bp located between the distal and proximal NrdR box pairs and doesn't contain NrdR boxes.

A

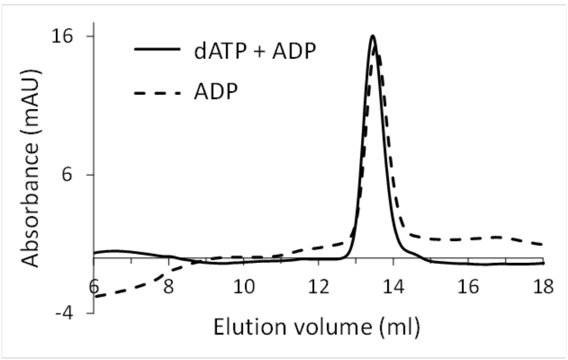

B

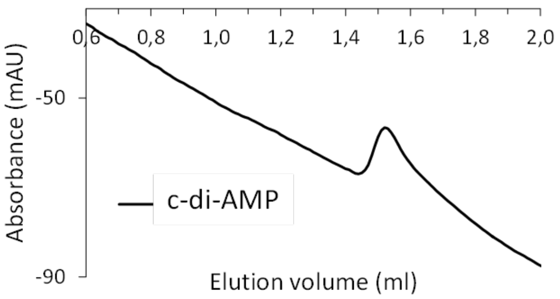

C

	ADP	dATP +ADP	c-di-AMP
Size (kDa)<br>(# of monomers)<br>Oligomeric state	80<br>(4.2)<br>tetramer	89<br>(4.7)<br>tetramer	80<br>(4.2)<br>tetramer

Supplementary figure 3. Size exclusion chromatography of LmoNrdR in the presence of different nucleotides. (A) Chromatography performed using Superdex 200 Increase 10/300 (24 ml column) in the presence of ADP (dashed) and dATP + ADP (solid line). 100 µl of 50 µM NrdR was injected. (B) Chromatography performed on Superdex 200 Increase 3.2/300 (2.4 ml column) in presence of c-di-AMP. 25 µl of 50 µM NrdR was injected. (C) summary of calculated molecular weights of LmoNrdR in presence of different effectors and its estimated oligomeric state. In parentheses is a calculated number of NrdR monomeric subunits (19 kDa).

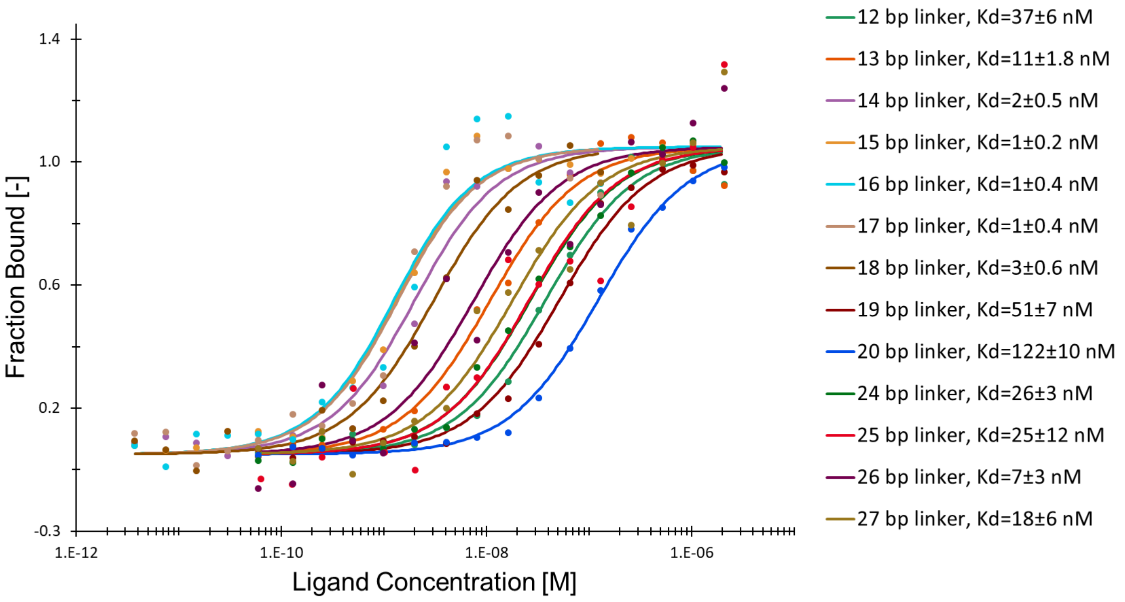

Supplementary figure 4. Binding of LmoNrdR to synthetic NrdR boxes with varying spacer length determined by MST. Plots of protein fraction bound vs. the concentration of ligand (NrdR) are shown. Lines represent fits of the data points using the K_D fit derived from the law of mass action. K_D and standard deviations (mean values ± SD) were calculated by the analysis software using fits from three individual titrations.

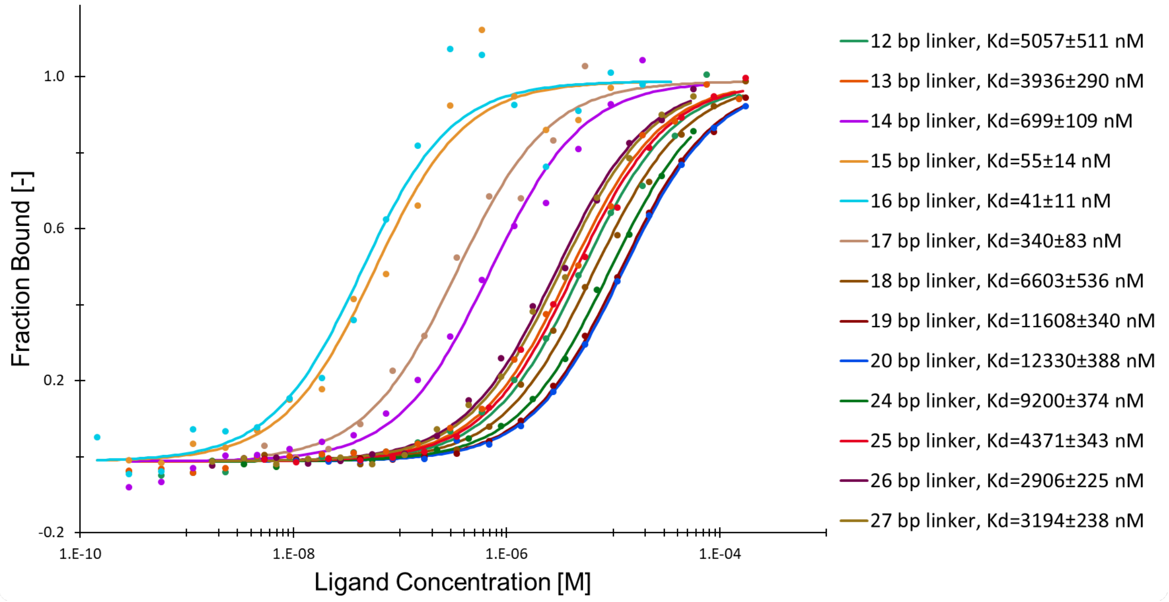

Supplementary figure 5. Binding of SpnNrdR to synthetic NrdR boxes with varying spacer length determined by MST. Plots of protein fraction bound vs. the concentration of ligand (NrdR) are shown. Lines represent fits of the data points using the K_D fit derived from the law of mass action. K_D and standard deviations (mean values ± SD) were calculated by the analysis software using fits from three individual titrations.

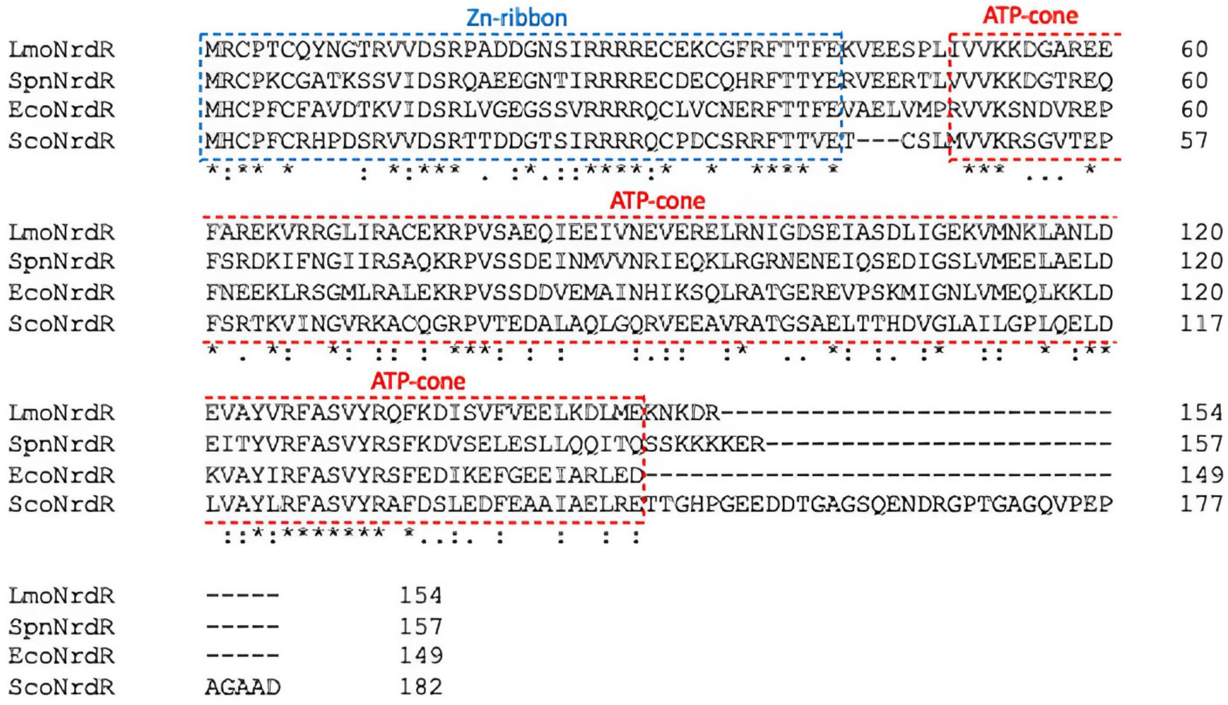

Supplementary figure 6. Sequence alignment of NrdR proteins from *L. monocytogenes* (LMRG_RS07775), *Streptococcus pneumoniae* (SP_1713), *E. coli* (b0413) and *Streptomyces coelicolor* (SCO5804).

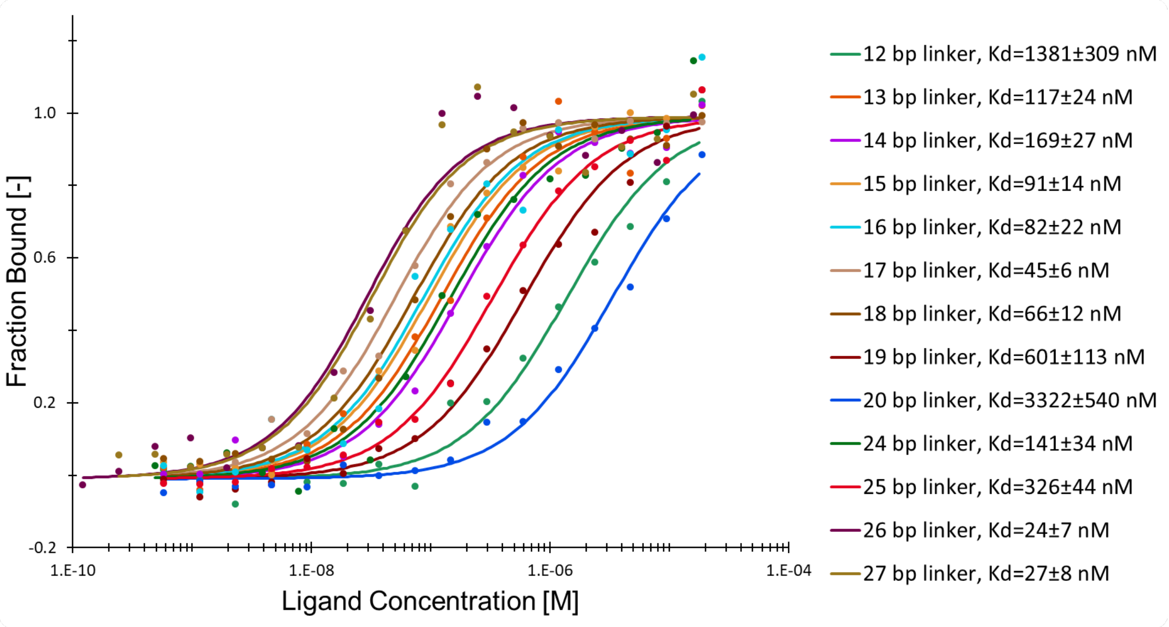

Supplementary figure 7. Binding of EcoNrdR to synthetic NrdR boxes with varying spacer length determined by MST. Plots of protein fraction bound vs. the concentration of ligand (NrdR) are shown. Lines represent fits of the data points using the K_D fit derived from the law of mass action. K_D and standard deviations (mean values ± SD) were calculated by the analysis software using fits from three individual titrations.

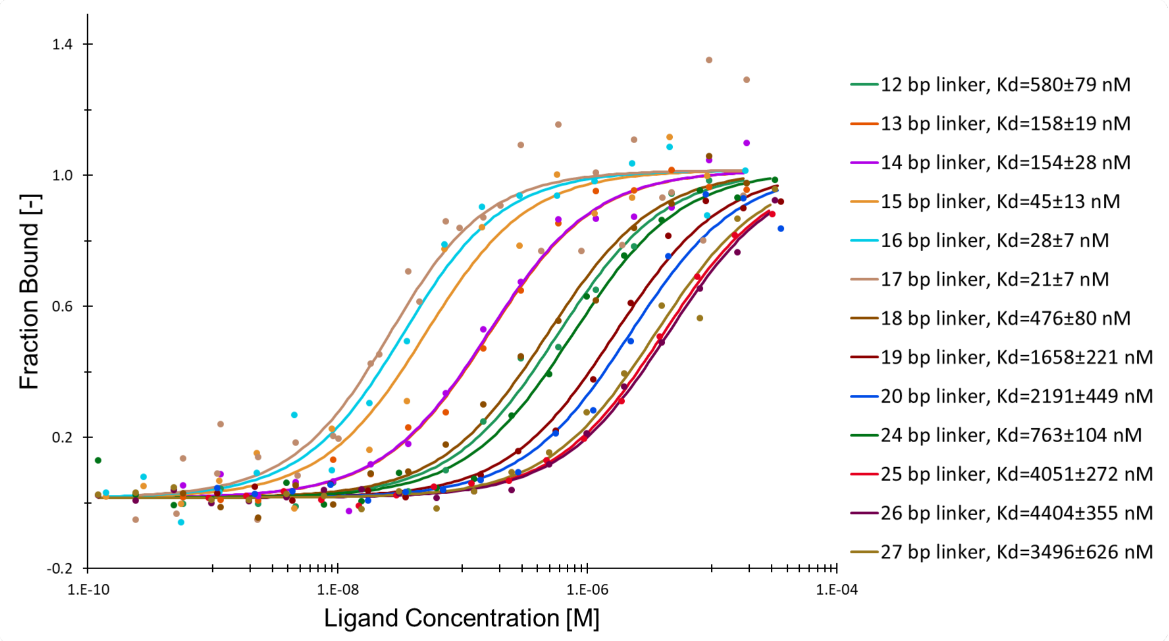

Supplementary figure 8. Binding of ScoNrdR to synthetic NrdR boxes with varying spacer length determined by MST. Plots of protein fraction bound vs. the concentration of ligand (NrdR) are shown. Lines represent fits of the data points using the K_D fit derived from the law of mass action. K_D and standard deviations (mean values ± SD) were calculated by the analysis software using fits from three individual titrations.

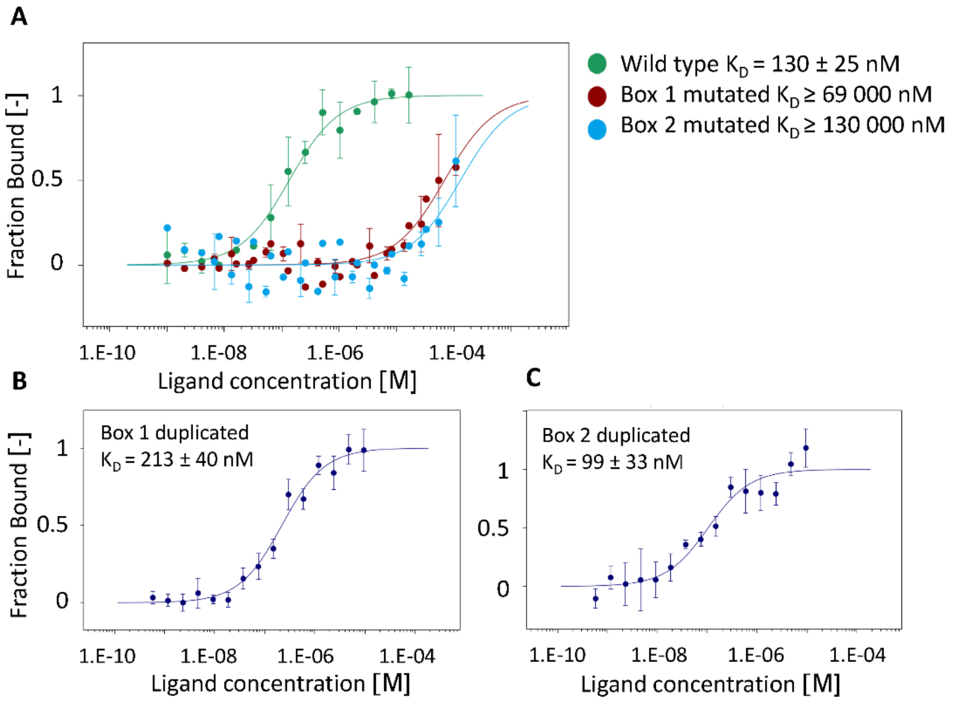

Supplementary figure 9. Binding of EcoNrdR to wild-type and mutated NrdR boxes derived from the *E. coli nrdHIEF* promoter region. (A) Wild-type NrdR binding site (green) and mutated fragments in which NrdR box 1 (red) or NrdR box 2 (blue) were mutated. (B, C) Fragments containing a duplication of NrdR box 1 or NrdR box 2, respectively. See Materials and Methods and Supplementary fig. 3 for details. Plots of protein fraction bound vs. the concentration of ligand (NrdR) are shown. Lines represent fits of the data points using the K_D fit derived from the law of mass action. K_D and standard deviations (mean values ± SD) were calculated by the analysis software using fits from three individual titrations.

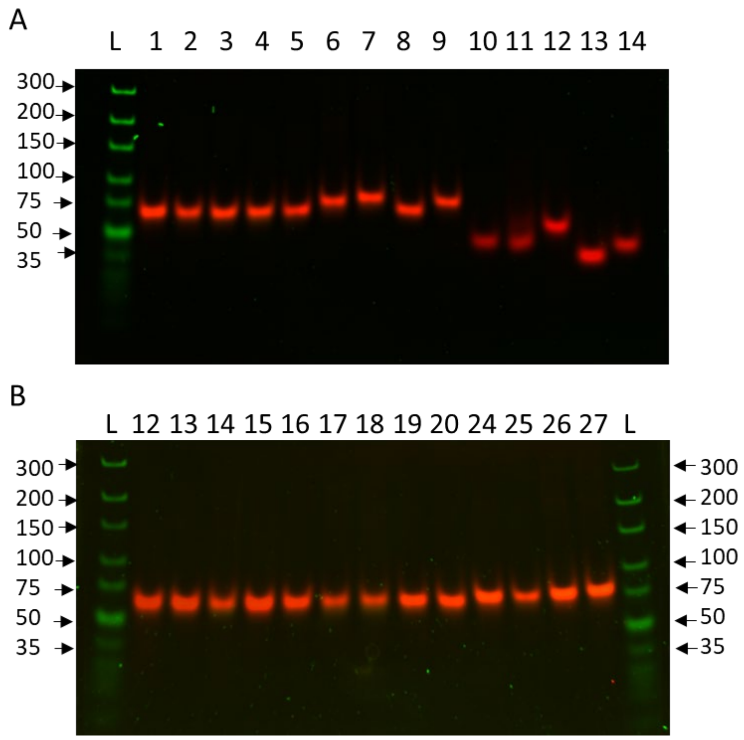

Supplementary figure 10: PAGE of annealed oligonucleotides used for MST. (A) L) DNA ladder; 1) LmoABdistal; 2) LmoABproximal; 3) LmoDG; 4) Negative control oligo; 5) SthAB; 6) SpnAB1,2; 7) SpnAB2,3; 8) SthDG; 9) SpnDG; 10) SthEF1,2_For; 11) SpnAB1,2_For); 12) SpnAB1,2_For; 13) SthDG_For; 14) SpnDG_For. “For” stands for single stranded sense oligo (negative control for annealing reaction). (B) synthetic oligonucleotides with different spacer lengths: L) DNA ladder; the numbers 12-27 correspond to the length of the spacer between NrdR boxes. 1 pmol double stranded oligonucleotide was loaded on 5% polyacrylamide gel in Tris/Borate/EDTA (TBE) buffer and ran at 70 V for ~90 minutes. Gene ruler Ultra low (Thermo Fischer Scientific) was used as DNA ladder.

Supplementary table 1. Oligonucleotides used for analyzing the effect of spacer length on NrdR binding. Red font indicates the spacer between the palindromic regions of NrdR boxes; underlining indicates position of NrdR boxes. Deleted nucleotides are shown in strike through lowercase gray while the added ones are shown in green color.

Spacer length (bp)	Sequence (sense oligo)
12	5′-Cy5-GCCAAACACAACATCTAGTGGTTGGATAG~~cgt~~GAGCACACAACATCTAGTGGACCTC-3′
13	5′-Cy5-GCCAAACACAACATCTAGTGGTTGGATAG~~cg~~TGAGCACACAACATCTAGTGGACCTC-3′
14	5′-Cy5-GCCAAACACAACATCTAGTGGTTGGATAGC~~g~~TGAGCACACAACATCTAGTGGACCTC-3′
15	5′-Cy5-GCCAAACACAACATCTAGTGGTTGGATAGCGTGAGCACACAACATCTAGTGGACCTC-3′
16	5′-Cy5-GCCAAACACAACATCTAGTGGTTGGATAGCGTGAAGCACACAACATCTAGTGGACCTC-3′
17	5′-Cy5-GCCAAACACAACATCTAGTGGTTGGATAGCGTGAAGGCACACAACATCTAGTGGACCTC-3′
18	5′-Cy5-GCCAAACACAACATCTAGTGGTTGGATAGCGTGAAGTGCACACAACATCTAGTGGACCTC-3′
19	5′-Cy5-GCCAAACACAACATCTAGTGGTTGGATAGCGTGAAGTAGCACACAACATCTAGTGGACCTC-3′
20	5′-Cy5-GCCAAACACAACATCTAGTGGTTGGATAGCGTGACAGTAGCACACAACATCTAGTGGACCTC-3′
24	5′-Cy5-GCCAAACACAACATCTAGTGGTTGGATAGCGTGAATTCACCTAGCACACAACATCTAGTGGACCTC-3′
25	5′-Cy5-GCCAAACACAACATCTAGTGGTTGGATAGCGTGAAGTTCACCTAGCACACAACATCTAGTGGACCTC-3′
26	5′-Cy5-GCCAAACACAACATCTAGTGGTTGGATAGCGTGAAGTTCCACCTAGCACACAACATCTAGTGGACCTC-3′
27	5′-Cy5-GCCAAACACAACATCTAGTGGTTGGATAGCGTGAAGTTACCACCTAGCACACAACATCTAGTGGACCTC-3′

Supplementary table 2. Oligonucleotides (antisense) used for analyzing the effect of spacer length on NrdR binding.

Spacer length (bp)	Sequence (antisense oligo)
12	5′-GAGGTCCACTAGATGTTGTGTGCTCCTATCCAACCACTAGATGTTGTGTTTGGC-3′
13	5′-GAGGTCCACTAGATGTTGTGTGCTCACTATCCAACCACTAGATGTTGTGTTTGGC-3′
14	5′-GAGGTCCACTAGATGTTGTGTGCTCAGCTATCCAACCACTAGATGTTGTGTTTGGC-3′
15	5′-GAGGTCCACTAGATGTTGTGTGCTCACGCTATCCAACCACTAGATGTTGTGTTTGGC-3′
16	5′-GAGGTCCACTAGATGTTGTGTGCTTCACGCTATCCAACCACTAGATGTTGTGTTTGGC-3′
17	5′-GAGGTCCACTAGATGTTGTGTGCCTTCACGCTATCCAACCACTAGATGTTGTGTTTGGC-3′
18	5′-GAGGTCCACTAGATGTTGTGTGCACTTCACGCTATCCAACCACTAGATGTTGTGTTTGGC-3′
19	5′-GAGGTCCACTAGATGTTGTGTGCTACTTCACGCTATCCAACCACTAGATGTTGTGTTTGGC-3′
20	5′-GAGGTCCACTAGATGTTGTGTGCTACTGTCACGCTATCCAACCACTAGATGTTGTGTTTGGC-3′
24	5′-GAGGTCCACTAGATGTTGTGTGCTAGGTGAATTCACGCTATCCAACCACTAGATGTTGTGTTTGGC-3′
25	5′-GAGGTCCACTAGATGTTGTGTGCTAGGTGAACTTCACGCTATCCAACCACTAGATGTTGTGTTTGGC-3′
26	5′-GAGGTCCACTAGATGTTGTGTGCTAGGTGGAACTTCACGCTATCCAACCACTAGATGTTGTGTTTGGC-3′
27	5′-GAGGTCCACTAGATGTTGTGTGCTAGGTGGTAACTTCACGCTATCCAACCACTAGATGTTGTGTTTGGC-3′

Supplementary table 3: Strain-specific oligonucleotides used in MST experiments. Underlining indicates position of the NrdR boxes. Grey highlighting indicates mutations made in the NrdR boxes.

MST oligonucleotide	sequence
Lmo_nrdAB_distal_sense	5′-Cy5-CTTAACTACTAAATATAGAAACTAGCGAAAAAATAGGCAC TAGAAGTAGTAGCATTC-3′
Lmo_nrdAB_distal_antisense	5′-Cy5-GAATGCTACTACTTCTAGTGCCTATTTTTTCGCTAGTTTCT ATATTTAGTAGTTAAG-3′
Lmo_nrdAB_proximal_sense	5′-Cy5-CCAGAGTACTATATCTTGCATTCACAATGAAAAAGTTATAC TATATATAGTATCGAAC-3′
Lmo_nrdAB_proximal_antisense	5′-GTTCGATACTATATATAGTATAACTTTTTCATTGTGAATGCAAG ATATAGTACTCTGG-3′
Lmo_nrdDG_sense	5′-Cy5-CAATAAAACTTCATCTTGTGTTATATTATTAAAGCAGACAC AATATATAGTTAGTGGG-3′
Lmo_nrdDG_antisense	5′-CCCACTAACTATATATTGTGTCTGCTTTAATAATATAACACAAG ATGAAGTTTTATTG-3′
Spn_nrdHEF_1,2_sense	5′-Cy5-TACAAACACTATATATTGTGTTTATATATAAAAACAACAAA AGAACACCACTATATTTAGTTCAAATC-3′
Spn_nrdHEF_1,2_antisense	5′-GATTTGAACTAAATATAGTGGTGTTCTTTTGTTGTTTTTATATA TAAACACAATATATAGTGTTTGTA-3′
Spn_nrdHEF_0,1_sense	5′-Cy5-TTTAAAAACAATTTATAGGATAATCATTGCTATTTCACAAT ACAAACACTATATATTGTGTTTATA-3′
Spn_nrdHEF_0,1_antisense	5′-TATAAACACAATATATAGTGTTTGTATTGTGAAATAGCAATGAT TATCCTATAAATTGTTTTTAAA-3′
Sth_nrdHEF_sense	5′-Cy5-TCAAAACACAAGATATAGGGTTGTGTTTCAATACTAAACA CAAAATATTGTATTCCTT-3′
Sth_nrdHEF_antisense	5′-AAGGAATACAATATTTTGTGTTTAGTATTGAAACACAACCCTA TATCTTGTGTTTTGA-3′
Spn_nrdDG_sense	5′-Cy5-AACCAAATCAATATCTTGTGTATTTTTAAAAATTTTAGGAT TTTTAACACAAGATATTGATTTTCCT-3′
Spn_nrdDG_antisense	5′- AGGAAAATCAATATCTTGTGTTAAAAATCCTAAAATTTTTAAAA ATACACAAGATATTGATTTGGTT-3′
Sth_nrdDG_sense	5′-Cy5-TCGAAAATCAATATATAGTGGTTGAGATAAAAATATACCAC AATATATTGATTTTCCT-3′
Sth_nrdDG_antisense	5′-AGGAAAATCAATATATTGTGGTATATTTTTATCTCAACCACTAT ATATTGATTTTCGA-3′
Negative_control_sense	5′-Cy5-GATTTGCTGAAAACCTTGTTGTAATCATTGTTTAGACACT TTTCGTAATAAAACCAG-3′
Negative_control_antisense	5′-CTGGTTTTATTACGAAAAGTGTCTAAACAATGATTACAACAAG GTTTTCAGCAAATC-3′
Eco_nrdHEF_sense	5′-Cy5- ACTATTTGCTATATATTGTGTGGTTGAATCTTTTTTCAACTAC ATCTAGTATCTCTG
Eco_nrdHEF_antisense	5′-CAGAGATACTAGATGTAGTTGAAAAAAGATTCAACCACACAAT ATATAGCAAATAGT-3′
Eco_nrdHEF_Box1_Mutated_ sense	5′-Cy5-ACTATTTATTATGGATTATGTGGTTGAATCTTTTTTCAACTAC ATCTAGTATCTCTG-3′
Eco_nrdHEF_Box1_Mutated_ antisense	5′-CAGAGATACTAGATGTAGTTGAAAAAAGATTCAACCACATAAT CCATAATAAATAGT-3′
Eco_nrdHEF_ Box2_Mutated_ sense	5′-Cy5-ACTATTTGCTATATATTGTGTGGTTGAATCTTTTTTGAATTAC GGCTAATATCTCTG-3′
Eco_nrdHEF_ Box2_Mutated_ antisense	5′-CAGAGATATTAGCCGTAATTCAAAAAAGATTCAACCACACAAT ATATAGCAAATAGT-3′
Eco_nrdHEF_Box1_Duplicated_ sense	5′-Cy5-GTCTCTTGCTATATATTGTGTGGTTGAATCTTTTTTTTGCTATA TATTGTGTCTCTG-3′
Eco_nrdHEF_Box1_Duplicated_ antisense	5′-CAGAGACACAATATATAGCAAAAAAAAGATTCAACCACACAATATA TAGCAAGAGAC-3′
Eco_nrdHEF_ Box2_Duplicated _sense	5′-Cy5-GTCTCCAACTACATCTAGTATGGTTGAATCTTTTTTCAACTACA TCTAGTATCTCTG-3′
Eco_nrdHEF_Box2_Duplicated_ antisense	5′-CAGAGATACTAGATGTAGTTGAAAAAAGATTCAACCATACTAGATGT AGTTGGAGAC-3′

Supplementary table 4. Sequence of NrdR boxes and the spacer between them in fragments used for reporter gene analysis. Underlining in the wild type fragment indicates position of the NrdR boxes. Mutations in the NrdR boxes are highlighted grey in the fragment where they were mutated *.

Spacer<br>length (bp)	Sequence
12	5′-TTGCTATATATTGTGTTGAATCTTTTTTCAACTACATCTAGTAT-3′
13	5′-TTGCTATATATTGTGTTTGAATCTTTTTTCAACTACATCTAGTAT-3′
14	5′-TTGCTATATATTGTGTGTTGAATCTTTTTTCAACTACATCTAGTAT-3′
15<br>(wild type)	5′-TTGCTATATATTGTGTGGTTGAATCTTTTTTCAACTACATCTAGTAT-3′
16	5′-TTGCTATATATTGTGTTGGTTGAATCTTTTTTCAACTACATCTAGTAT-3′
17	5′-TTGCTATATATTGTGTTTGGTTGAATCTTTTTTCAACTACATCTAGTAT-3′
18	5′-TTGCTATATATTGTGTTGTGGTTGAATCTTTTTTCAACTACATCTAGTAT-3′
19	5′-TTGCTATATATTGTGTTGTTGGTTGAATCTTTTTTCAACTACATCTAGTAT-3′
20	5′-TTGCTATATATTGTGTTGGTTGGTTGAATCTTTTTTCAACTACATCTAGTAT-3′
24	5′-TTGCTATATATTGTGTTGGTTGTTTGGTTGAATCTTTTTTCAACTACATCTAGTAT-3′
25	5′-TTGCTATATATTGTGTTGGTTTGTTTGGTTGAATCTTTTTTCAACTACATCTAGTAT-3′
26	5′-TTGCTATATATTGTGTTGGTTTGATTTGGTTGAATCTTTTTTCAACTACATCTAGTAT-3′
27	5′-TTGCTATATATTGTGTTGGTTTTGATTTGGTTGAATCTTTTTTCAACTACATCTAGTAT -3′
Mutated<br>NrdR boxes	5-‘TTGTTATATATTATGTGGTTGAATCTTTTTTCAATTACATCTAATAT-3′

* The conserved base pairs at the positions 4 and 13 in NrdR boxes are crucial for NrdR DNA recognition and their mutation results in at least 350-fold reduction in binding affinity (Rozman Grinberg *et al. Nat Commun* **13**, 2700 (2022).
